# Dual-targeting peptides@PMO, a mimetic to the pro-apoptotic protein Smac/DIABLO for selective activation of apoptosis in cancer cells

**DOI:** 10.3389/fphar.2023.1237478

**Published:** 2023-08-29

**Authors:** Eros Di Giorgio, Annalisa Ferino, Weizhe Huang, Sigrid Simonetti, Luigi Xodo, Rossella De Marco

**Affiliations:** ^1^ Department of Medicine, University of Udine, Udine, Italy; ^2^ Department of Agricultural, Food, Environmental and Animal Sciences (Di4A), University of Udine, Udine, Italy

**Keywords:** Smac mimetics, nanoparticles, caspases, oxaliplatin, cervical cancer, colorectal cancer, integrin αvβ3

## Abstract

The refractoriness of tumor cells to apoptosis represents the main mechanism of resistance to chemotherapy. Smac/DIABLO mimetics proved to be effective in overcoming cancer-acquired resistance to apoptosis as a consequence of overexpression of the anti-apoptotic proteins XIAP, cIAP1, and cIAP2. In this work, we describe a dual-targeting peptide capable of selectively activating apoptosis in cancer cells. The complex consists of a fluorescent periodic mesoporous organosilica nanoparticle that carries the short sequences of Smac/DIABLO bound to the αvβ3–integrin ligand. The dual-targeting peptide @PMO shows significantly higher toxicity in αvβ3-positive HeLa cells with respect to αvβ3-negative Ht29 cells. @PMO exhibited synergistic effects in combination with oxaliplatin in a panel of αvβ3-positive cancer cells, while its toxicity is overcome by XIAP overexpression or integrin β3 silencing. The successful uptake of the molecule by αvβ3-positive cells makes @PMO promising for the re-sensitization to apoptosis of many cancer types.

## 1 Introduction

**FIGURE 1:**
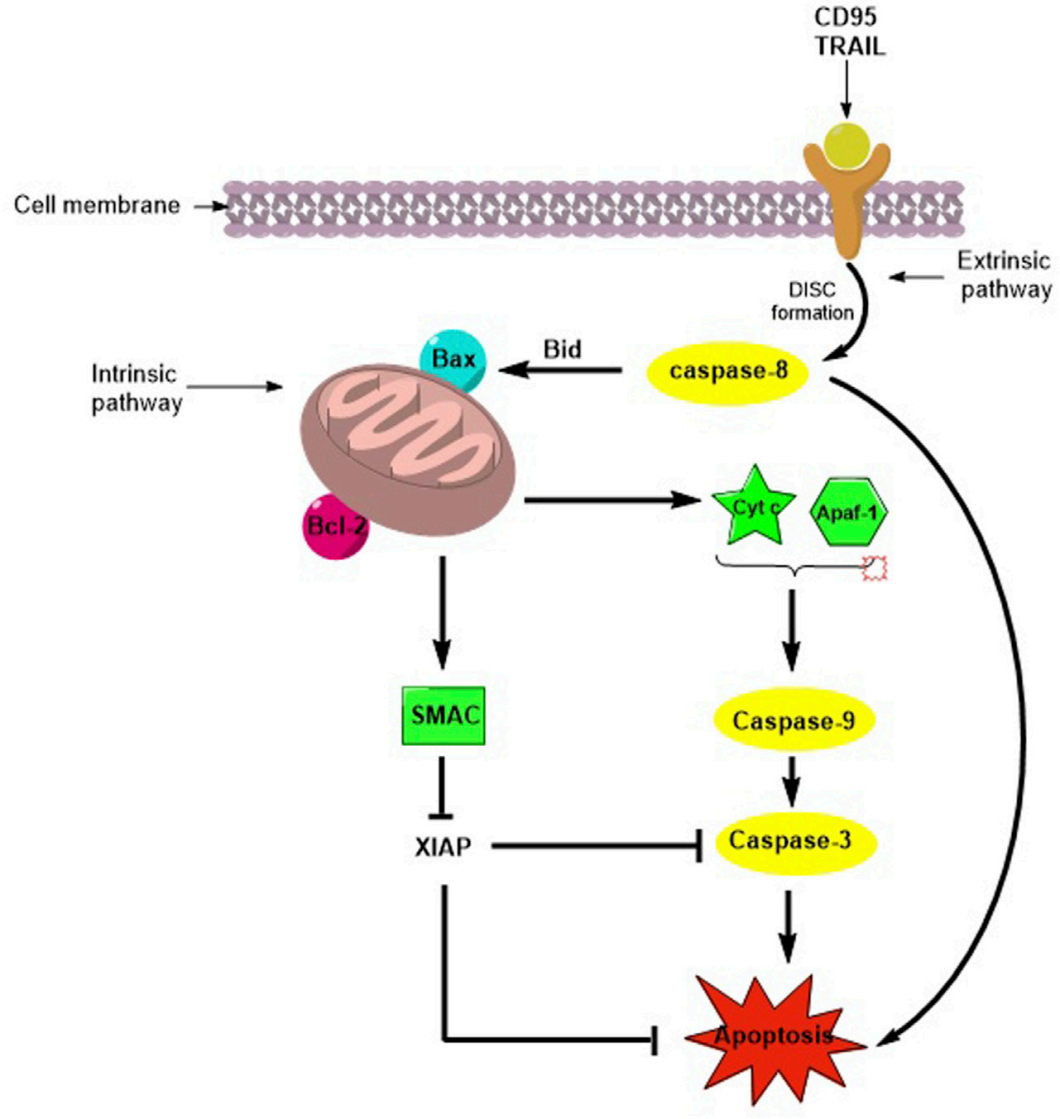
Schematic representation of apoptosis activation by extrinsic and intrinsic pathways.

Tumor cells can escape apoptosis, which is the main mechanism of resistance to chemotherapy (Dogan et al. 2021). Apoptosis or programmed cell death (PCD) is a natural mechanism that maintains the turnover of healthy cells in tissues. The pathways involved in PCD are the extrinsic (or death receptor pathway) and the intrinsic (or mitochondrial pathway) pathways, and both mechanisms lead to the activation of Cysteine-ASPartic acid-specific ProteASEs (caspases), a family of cysteine proteases that mediate apoptosis and a variety of other physiological and pathological processes (Degterev et al., 2003). When apoptosis is activated via the extrinsic pathway by natural ligands, death receptor engagement and DISC complex assembly lead to the activation of caspase-8, which directly activates caspase-3 and initiates apoptosis. The intrinsic pathway is regulated by the B-cell lymphoma-2 (Bcl-2) superfamily and induces the release of pro-apoptotic factors from the mitochondria. Cytochrome-c and Apaf-1 promote caspase-9 activation, while the second mitochondria-derived activator of caspase/direct inhibitor of apoptosis proteins (IAP) binding protein with low PI (Smac/DIABLO) binds inhibitor apoptotic protein (IAP), which is essential for regulating caspase-3, -7, and -9 activity, ultimately enhancing caspase activation (Fulda, 2009a; Kocab and Duckett, 2015) (Figure 1). IAPs are a family of anti-apoptotic proteins that include the following: X-linked (XIAP), cellular (cIAP1 and cIAP2), neuronal (NIAP), testis-specific (Ts-IAP), Bir-ubiquitin-conjugating enzyme (BRUCE), survivin, and livin. They contain zinc finger Baculovirus IAP Repeat (BIR) domains, which are responsible for regulating apoptosis in cells (Huang et al., 2001). Overexpression of IAPs is associated with resistance to chemo- and radiotherapies in several cancers (Hundsdoerfer et al., 2010), and direct inhibition of IAPs by antagonists such as Smac mimetics (SMs), which are important to improve the metabolic stability and bioavailability of the native peptide (Gentilucci et al., 2010), induces apoptosis and contributes to overcoming drug resistance (Fulda, 2009b).

There are several approaches for inhibiting the overexpression of IAPs: a) by downregulating their expression when apoptotic stimuli are present; b) by upregulating natural proapoptotic proteins; c) by directly inhibiting the action of IAPs on caspases (LaCasse et al., 2008). All of these approaches could be used to re-sensitize cancer cells to chemotherapeutic agents through a synergistic effect with combination therapy. Dual-targeting approaches consist in the usage of compounds that contain two or more ligands that interact with two or more targets (Morphy and Rankovic, 2005). For example, the synergistic action of SMs with gemcitabine, a chemotherapeutic agent used to treat pancreatic cancer, results in nuclear factor (NF)-κB-mediated caspase activation and subsequent cell death (Stadel et al., 2011). The combination of LCL161, a Smac mimetic, with oncolytic rhabdoviruses (vesicular stomatitis viruses) caused synergistic bystander cell death in cancer cells and induced significant tumor regression and a durable response *in vivo* (Beug et al., 2014).

The target therapy approach allows overcoming the need for selectivity toward cancer cells by conventional chemotherapeutic drugs. Conjugation with peptide ligands capable of recognizing receptors overexpressed on tumor cells ensures selectivity and improves cellular uptake (Ross et al., 2004). In this context, integrins represent an excellent target for cancer cells. Integrins are heterodimers of proteins present on the cell surface. In particular, integrins αvβ3 and αvβ1 are involved in many cellular processes and regulate the initiation, progression, and metastasis of solid tumors by binding the tripeptide arginine–glycine–aspartic acid (RGD) present on fibronectin and vitronectin, their natural ligands (De Marco et al., 2016). In that regard, RGD mimetic was bound with SMs to form the trans-Cyclo [ABN-RGD]-ABD and tested on MDA-MB-231 and IGROV-1 cells. The conjugate, trans-Cyclo[ABN-RGD]-ABD, showed a cytotoxic activity of 20.5 μM on MDA-MB-231 and 11.5 μM on IGROV-1 (Mingozzi et al., 2014).

Use of nanoparticles (NPs) as nanocarriers is an emerging approach. The potential of nanocarriers is that they improve drug efficacy, solubility, and stability, thereby reducing drug toxicity (Alexis et al. 2008). Nanoparticles exhibit a prolonged circulation time and can be used in both active and passive targeting strategies, increasing drug/peptide concentration in tumor cells while avoiding toxicity in healthy cells (Allen, 2002). Passive targeting takes advantage of features in tumor biology that allow nanocarriers to accumulate in a tumor due to enhanced permeability and retention (EPR) (Maeda, 2001). Active approaches present chemotherapeutics bound with recognition molecules, for example, membrane receptors, conjugated onto nanocarriers (Dadwal et al., 2018). In this context, SMAC-N7 was conjugated to nanoparticles, resulting in the inhibition of cell proliferation and apoptosis in breast cancer cells (MDA-MB -231) and non-small lung cancer cells (H460). SMAC-N7-NPs induce activation of pro-caspase-3, downregulation of Bcl-2, and upregulation of Bax with an apoptosis effect in both MDA-MB -231 and H460 cancer cells (Li, et al., 2015).

Periodic mesoporous organosilicas (PMOs) are versatile nanoparticles. They were first synthesized in 1999 by Ozin, Stein, and Inagaki. PMOs are synthesized by sol–gel self-assembly processes of silsesquioxane of the type (EtO)_3_Si-R-Si-(OEt)_3_, where the organic group -R- is localized within the channel walls (Asefa et al., 1999; Inagaki et al., 1999; Melde et al., 1999). They exhibit interesting properties such as a high degree of order; uniform pore size that can be tuned to accommodate drugs of different sizes; high biocompatibility; a large surface area that can be functionalized with drugs or peptides, enabling drug delivery and nanocarrier applications (Hatton et al., 2005); and intrinsic electrocatalytic activity that improves selectivity, detection, and long-term durability of sensors (Aissa et al., 2023).

In this work, we connected the cyclic RGD with the short sequence Alanine-Valine-Proline-Isoleucine (AVPI) of Smac/DIABLO, which is responsible for the cytotoxic activity, via a lysine bridge and coupled it to nanoparticles. The system showed high uptake, attributed to the presence of the fibronectin competitor that binds the integrin αvβ3, allowing it to selectively interact and pass through the cell membrane of cancer cells (Desgrosellier and Cheresh, 2010). At the same time, the presence of AVPI activates the intrinsic pathway by inhibiting XIAP and activating apoptosis.

## 2 Material and methods

The chemical reagents and the solvents were purchased from Sigma Aldrich (Italy) and used without further purification. The reactions were carried out in an unmodified domestic microwave oven (Candy, CMG2071DS, input 1200 W, output 700 W, and frequency 2,450 MHz).

## 3 Chemistry

### 3.1 General procedure for solid-phase peptide synthesis

The 2-chlorotrityl chloride resin (Cl-Trt-resin) preloaded with amino acid (1.1 mmol) was swollen in DCM (5 mL). The 9-fluorenylmethoxycarbonyl (Fmoc) amino acid (3.3 mmol) was dissolved in DMF (5 mL), and 1-hydroxybenzotriazole (HOBt) (3.3 mmol) and *N,N*′-dicyclohexylcarbodiimide (DCC) (3.3 mmol) were added as coupling reagents. The mixture was stirred for 3 h. The resin was washed three times with the following solvents: DCM (5 mL), MeOH (5 mL), and DMF (5 mL). The peptide bond formation was evaluated using the Kaiser test. The Fmoc protective group was removed by a solution of piperidine (20%) in DMF (5 mL) for 45 min at room temperature. After filtering and washing with DCM, MeOH, and DMF, the complete Fmoc deprotection was evaluated using the Kaiser test. The steps of coupling and deprotection were repeated for each amino acid.

### 3.2 Peptide cleavage *Fmoc Ala-Val-Pro-Ile-Gly-Lys-OH* **(SS24)**

The half peptidyl resin **SS22** was treated with a solution of trifluoroacetic acid (TFA) and triisopropylsilane/water/phenol (TIPS/H_2_O/PhOH (9:0.5:0.5 and v/v 20 mL)) and left to be stirred for 2.5 h at room temperature. After that, the suspension was filtered and washed with ether (Et_2_O)/TFA (95:5 and v/v 10 mL), Et_2_O, and methanol. The combined filtrates were evaporated under vacuum. The residual oil was treated with ice-cold ether to yield the product **SS24** (325 mg, 0.40 mmol, 36.4%) as a white precipitate. The solid was recovered by centrifugation. Purities were determined to be >95% by analytical RP HPLC and elemental analysis (for the conditions, see General Methods). The identity of the compounds was confirmed by ^1^H NMR, 2D NMR, and ESI MS analyses.

^1^H NMR (400 MHz, [D6] DMSO) δ: 0.86 (d, J = 6.6 Hz, 6H, IleCH_3_ + ValCH_3_), 0.88 (d, J = 6.6 Hz, 3H, IleCH_3_), 0.90 (d, J = 6.6 Hz, 3H, ValCH_3_), 1.20 (d, J = 3.5 Hz, 3H, AlaCH_3_), 1.35–1.40 (m, 4H, LysHγ + IleCH_2_), 1.45–1.53 (m, 4H LysHβ + LysHδ), 1.70–1.74 (m, 4H, ValHβ + ProHβ + IleΗβ), 2.71–2.76 (m, 4H, ProHγ + LysHε), 3.30–3.33 (m, 2H, ProHδ), 3.76 (dd, J = 6.0. 15.0 Hz, 2H, GlyHα), 4.11 (t, J = 7.36 Hz, 2H, AlaHα + ValHα), 4.27–4.30 (m, 2H, LysHα + IleHα), 4.40–4.50 (m, 4H, ProHα+ FmocCH_2_,CH), 7.34 (t, J = 7.16 Hz, 2H, ArFmoc), 7.42 (t, J = 7.56 Hz, 2H, ArFmoc), 7.51 (d, J = 8.52 Hz, 1H, AlaNH), 7.86 (d, J = 7.36 Hz, 1H, ValNH), 7.90–8.10 (m, 6H, ArFmoc + IleNH + LysNH), 8.14 ( t, J = 5.60 Hz, 1H, GlyNH). ES-MS m/z [M+2H]^2+^/2 and [M+H]^+^found: 403.7 and 806.2, calculated: 806.4 calculated for C_42_H_59_N_7_O_9_. Elementary analysis for C_42_H_59_N_7_O_9_ calculated: C 62.51, H 7.49, N 12.15, found: C 62.32, H 7.62, N 12.02.

### 3.3 Peptide cleavage FmocAla-Val-Pro-Ile-Gly-Lys(Boc)-OH and Arg(Pbf)-Gly-Asp(OtBu)phe-Lys(Cbz)-OH

Peptide cleavage *Fmoc Ala-Val-Pro-Ile-Gly-Lys(Boc)-OH* and *Arg(Pbf)-Gly-Asp(OtBu)-phe-Lys*(*Cbz*)*OH* **(SS23 and SS16):** the peptidyl resin was treated with a solution of trifluoroethanol/acetic acid/dichlomethane (TFE/CH_3_COOH/DCM (1:1:4 and v/v 20 mL)) and allowed to stir for 90 min at room temperature. After that, the suspension was filtered and washed with Et_2_O, DCM, and methanol. The solvents were collected and evaporated under vacuum. The residual oil was treated with ice-cold ether to yield products **SS23** (325 mg, 0.40 mmol, and 32.7%) and **SS16** (650 mg, 0.61 mmol, and 55.5%) as white precipitates. The solids were recovered by centrifugation.

^*1*^ *H NMR FmocAla-Val-Pro-Gly-Lys*(*Boc*)*OH* (**SS23**) (400 MHz, [D6]DMSO) δ: 0.86 (d, J = 6.6 Hz, 6H, IleCH_3_ + ValCH_3_), 0.88 (d, J = 6.6 Hz, 3H, IleCH_3_) 0.90 (d, J = 6.6 Hz, 3H, ValCH_3_), 1.20 (d, J = 3.5 Hz, 3H, AlaCH_3_), 1.35–1.40 (m, 3H, LysHγ + IleCH_2_), 1.43 (s, 9H, Boc), 1.45–1.53 (m, 4H LysHβ + LysHδ), 1.70–1.74 (m, 4H, ValHβ + ProHβ + IleΗβ), 2.71–2.76 (m, 4H, ProHγ + LysHε), 3.30–3.33 (m, 2H, ProHδ), 3.76 (dd, J = 6.0. 15.0 Hz, 2H, GlyHα), 4.11 (t, J = 7.36 Hz, 2H, AlaHα + ValHα), 4.27–4.30 (m, 2H, LysHα + IleHα), 4.40–4.50 (m, 4H, ProHα+ FmocCH_2_,CH), 5.60–5.65 (m, 1H, LysNHBoc), 7.34 (t, J = 7.16 Hz, 2H, ArFmoc), 7.42 (t, J = 7.56 Hz, 2H, ArFmoc), 7.51 (d, J = 8.52 Hz, 1H, AlaNH), 7.86 (d, J = 7.36 Hz, 1H, ValNH), 7.90–8.10 (m, 6H, ArFmoc + IleNH + LysNH), 8.14 ( t, J = 5.60 Hz, 1H, GlyNH). ES-MS m/z found: 805.5 [M-100] and 928.5 [M+Na], calculated: 906.5 calculated for C_47_H_67_N_7_O_11_. Elementary analysis for C_47_H_67_N_7_O_11_ calculated: C 62.30, H 7.45, N 10.82, found: C 62.49, H 7.32, N 10.94.

### 3.4 Head-to-tail cyclization of linear peptide (**SS21**)

The linear peptide **SS16** (0.6 mmol) was solubilized in DMF (5 mL) and added drop by drop using a syringe pump to a mixture formed by diphenyl phosphoryl azide (DPPA) (1.8 mmol) and NaHCO_3_ (12 mmol) in DMF (10 mL) for 12 h at room temperature. After the reaction time, the solvent was removed under high vacuum. The solid was dissolved in 30 mL of ethyl acetate and washed with a solution of HCl 1 M (5 mL), a saturated solution of NaHCO_3_ (5 mL), and brine (5 mL). The solvent was removed under vacuum, and the crude product was purified by column chromatography (EtOAc- > EtOAc/MeOH (95:5, v/v)) to yield the final product **SS21** (550 mg, 0.53 mmol, and 88.3%). ES-MS m/z found for C_52_H_71_N_9_O_12_S 1046.2 [M+H]^+^ calculated: 1046.5.

### 3.5 *Cbz deprotection* (**SS25**)

Peptide **SS21** was solubilized in *i*PrOH in a flask, and HCOONH_4_ (4 equiv.) and Pd/C 10% (5% in weight with respect to the peptide) were added. The solution was irradiated in a microwave at 600 W for three cycles of 30 s each. The reaction was monitored by TLC under the disappearance of the starting material.

### 3.6 *Synthesis of dual-targeting peptide (DTP)* (**SS30**)

The Fmoc linear peptide **SS23** (0.14 mmol) was dissolved in DCM/DMF (3:1), HOBt (0.16 mmol) and 2-(1H-benzotriazole-1-yl)-1,1,3,3-tetramethylaminium tetrafluoroborate (TBTU) (0.16 mmol) were added to it. After 10 min, the cyclic peptide **SS25** (0.15 mmol) and *N,N*-diisopropylethylamine (DIPEA) were added (0.30 mmol) and stirred at room temperature. After 3 h, the solvent was removed under vacuum. The residual oil was solubilized with 30 mL of ethyl acetate and washed with HCl 1 M (5 mL) and a saturated solution of NaHCO_3_ (5 mL). The solvent was evaporated under vacuum, and the crude product was purified by column chromatography (EtOAc- > EtOAc/MeOH (80:20, v/v)) to yield the final product **SS30** (150 mg, 0.087 mmol, 62%) as colorless oil. ES-MS m/z found for C_91_H_130_N_16_O_20_S 1698.9 [M-100] and 1821.9 [M+Na]^+^calculated: 1799.9.

### 3.7 Procedure for final deprotection **(SS27 and SS33)**

Peptides **SS25** (0.50 mmol) and **SS30** (0.087 mmol) were dissolved in a mixture formed by TFA, triisopropylsilane, and water (90:5:5 v/v/v and 20 mL). The final solution was stirred for 1 h. After removing the solvent, the products were triturated in ether and recovered by centrifugation to yield the final products **SS27** and **SS33** as TFA salt and used without further purification. Purities were determined to be >95% by analytical RP-HPLC and elemental analysis (for the conditions, see *General Methods*). The identity of the compounds was confirmed by ^1^H NMR, 2D gCOSY, and ESI MS analyses.

#### 3.7.1 ^*1*^ *H NMR cyclic[RGDfK]* **(SS27)**

^1^H NMR cyclic[RGDfK] (400 MHz, [D6]DMSO) δ: 0.70–0.90 (m, 2H, LysHγ), 1.42–1.65 (m, 7H, LysHβ + LysHδ + ArgHβ + ArgHγ), 1.70–1.72 (m, 1H, ArgHβ), 2.38 (dd, J = 6.62, 16.35 Hz, 1H, AspHβ), 2.67–2.70 (m, 1H, AspHβ), 2.79–2.81 (m, 2H, LysHε), 2.83–2.85 (m, 1H, PheHβ), 3.07–3.10 (m, 1H, PheHβ), 3.25 (dd, J = 6.0, 14.4 Hz, 2H, ArgHδ), 3.94–3.96 (m, 2H, GlyHα), 4.04 (dd, J = 7.8, 15.1 Hz, 1H, LysHα), 4.13–4.18 (m, 1H, ArgHα), 4.43–4.45 m, 1H, PheHα), 4.63–4.65 (m, 1H, AspHα), 7.15–7.26 (m, 5H, PheArH), 7.34 (d, J = 9.6 Hz, 1H, ArgNH), 7.58 (bd, 1H, PheNH), 7.63 (d, J = 8.4 Hz, 1H, AspNH), 7.67–7.70 (m, 1H, LysNH), and 8.06–8.10 (bt, 1H, GlyNH). 280 mg, 0.46 mmol, 92%. ES-MS m/z found 604.5 [M+1], calculated: 604.3 calculated for C_27_H_41_N_9_O_7_. Elementary analysis for C_27_H_41_N_9_O_7_ calculated: C 53.72, H 6.85, and N 20.88, found: C 53.89, H 6.74, and N 21.10.

#### 3.7.2 ^*1*^ *H NMR Fmoc-AVPIGK@c[RGDfK]* (DTP) **(SS33)**

^1^H NMR (400 MHz, [D6]DMSO) δ: 0.75–0.87 (m, 3H, ValMe), 0.88–0.95 (m, 7H, ValMe + LysHγ_x4_), 1.12 (d, J = 6.8 Hz, 6H, IleMe_1,2_), 1.33–1.39 (m, 3H, AlaMe), 1.46–1.53 (m, 5H, IleCH_2_ + ArgHγ + LysHδ_x2_), 1.54–1.64 (m, 4H, ArgHγ + LysHδ_x2_), 1.90–2.02 (m, 10H, ValHβ + AlaHα + ProHβ + ProHγ + LysHβ_x4_), 2.66–2.70 (m, 2H, ArgHδ_x2_), 2.81–2.91 (m, 1H, AspHβ), 2.92–3.03 (m, 1H, AspHβ), 3.24–3.30 (m, 1H, IleHβ), 3.43–3.46 (m, 6H, PheHβ + LysHε_x4_), 3.53–3.62 (m, 2H, ProHδ), 3.64–3.75 (m, 4H, GlyHα), 4.00–4.05 (m, 1H, IleHα), 4.07–4.23 (m, 3H, ArgHα + LysHα_x2_), 4.30–4.40 (m, 6H, PheHα + ValHα + ProHα + FmocCH,CH_2_), 4.41–4.47 (m, 1H, AspHα), 6.71–6.76 (m, 1H, AlaNH), 6.93–7.03 (m, 5H, PheHAr + FmocHAr), 7.39–7.54 (m, 6H, PheHAr + FmocHAr), 7.15–7.27 (m, 2H, FmocHAr), 7.81–7.92 (m, 3H, ArgNH + LysNH_x2_), 7.96 (d, J = 9.1 Hz, 2H, PheNH + ValNH), and 8.09–8.17 (m, 3H, AspNH + GlyNH_1,2_). ES-MS m/z and found: 696.9 [M+2H]^2+^/2 and 1414.75 [M+Na]^+^, calculated: 1392.75 calculated for C_69_H_98_N_16_O_15_. Elementary analysis for C_69_H_98_N_16_O_15_ calculated: C 59.51, H 7.17, and N 16.09, found: C 59.32, H 7.05, and N 16.26.

### 3.8 *Synthesis of PMO* **(SS4)**

Hexadecyltrimethylammonium bromide (CTAB) (1.33 mol) was dissolved in H_2_O/ethanol (90 mL and 33 mL) and a 32 wt% ammonia (1.2 mL) solution. The reaction mixture was stirred at room temperature for 1 h before the addition of 1,2-bis(trimethoxysilyl)ethane (BTME) (0.005 mol). The aforementioned reaction mixture was continuously stirred for an additional time of 48 h at room temperature. The CTAB mesoporous template was removed by stirring the sample in ethanol (50 mL) with a 32 wt% aqueous solution of HCl (1.5 gr) at 50°C for 6 h. The resulting solid was recovered by centrifugation (6,000 rpm, 20 min), washed with ethanol three times, and dried at 60°C in vacuum (Motealleh et al., 2019).

### 3.9 *Synthesis of* ^*IR-780*^ *PMO* **(SS7)**

IR-780 (10 mg) was dissolved in dimethylsulfoxide (DMSO) (5 mL) and sonicated to complete dissolution of the dye. Then, the mixture was added drop by drop to 10 mL of water containing PMO (100 mg). The solution was stirred, in the dark, overnight and at room temperature. After this, the PMO was washed thrice with water, and the nanoparticles were recovered by centrifugation and left to dry at room temperature (Ma et al., 2020).

### 3.10 *Synthesis of* ^*IR-780*^ *PMO-ICPTES* **(SS14)**

To functionalize the ^IR-780^PMO-OH, we coated them with 3-isocyanatopropyltrymethoxy silane (ICPTES) and then with peptides. For ICPTES coating, ^IR-780^PMO-OH (100 mg) was suspended in toluene (5 mL) and triethylamine (TEA, 0.4 mL) and sonicated for 15 min. After that, ICPTES was added (1.6 mL). The reaction mixture was stirred overnight at room temperature. The nanoparticles were recovered by centrifugation and washed thrice with toluene.

### 3.11 *General procedure of synthesis of Peptides@* ^*IR-780*^ *PMO* **(SS28, SS35, and SS36)**

Peptides (20 mg) were dissolved in DMSO (1 mL) and TEA (120 μL) was added. The reaction mixtures were left sonicated for 10 min and then added to the PMO (20 mg). After stirring overnight at room temperature, the nanoparticles were washed twice with water and ethanol and recovered by centrifugation. PMO was left to dry under vacuum at 60°C.

### 3.12 *General procedure Fmoc cleavage from peptides@* ^*IR-780*^ *PMO* **(SS35 and SS36)**

Fmoc-peptides@PMO were dissolved in a solution of 20% piperidine in DMSO. The reaction mixtures were sonicated for 10 min and stirred for 40 min. After that, the nanoparticles were washed thrice with ethanol and recovered by centrifugation. The step of deprotection was repeated for 25 min. The PMOs were recovered by centrifugation, then washed thrice with ethanol, and dried at room temperature.

## 4 Characterization studies

### 4.1 Exact mass, elemental analysis, and nuclear magnetic resonance

Analytical RP HPLC was carried out on a C18 column (100 mm × 3 mm, particle size 3 μm, pore size 110 Å); DAD 210/246 nm, mobile phase from 9:1 H_2_O/CH_3_CN/0.1% TFA to 2:8 H_2_O/CH_3_CN/0.1% TFA in 20 min, flow rate 0.5 mL min^−1^. The exact mass was determined using a QTOF apparatus. The products were purified by flash chromatography, which was performed with silica gel (particle size: 40–60 µm) obtained from Merck. The purity was determined to be ≥ 95% by analytical RP HPLC, exact mass, and elemental analysis. Elemental analysis was performed with an EACE 1110 CHNS/O analyzer (Thermo Fisher, United States of America). NMR spectra were recorded by Bruker at 400 MHz in 8:2 DMSO–d_6_/H_2_O (water presaturation) and ^13^C NMR spectra at 100 MHz. Two-dimensional methods (HSQC; COSY) were used to support and confirm the assignment. The chemical shift δ is reported in ppm relative to the residual proton signal of the solvent: DMSO-d6 2.50 ppm (^1^H), 39.52 ppm (^13^C).

### 4.2 Average particle size (DLS) and scanning electron microscopy (SEM)

The nanoparticles were analyzed through dynamic light scattering (DLS) measurements performed with a Zetasizer Nano ZS (Malvern), He–Ne laser 633 nm, Max 4 mW, using polystyrene cuvettes (optical path length 1 cm). PMOs were dispersed in distilled water and sonicated for 20 min. The measurements were conducted at 20°C. Scanning electron microscopy, specifically the JSM-7610 FPLUS model by JEOL in Udine, Italy, was used to acquire high-resolution images of PMO nanoparticles. All images were collected using different magnifications.

### 4.3 Determination of PMOs peptide functionalization

To quantify the amounts of peptides on the PMO, a Varian Cary 50 bio benchtop spectrophotometer in the range of 200–800 nm with a 1-nm step was used (Faura et al. 2021). The calibration curves were determined by preparing five solutions at known concentrations of Phenylalanine and Fmoc-Alanine used as standards. Briefly, a stock solution of 5 mM Phe in absolute ethanol was diluted to the final concentrations of 0.2 mM, 0.5 mM, 1 m, 2 mM, and 5 mM. A standard solution of 52.7 μM Fmoc-Alanine (Ala) in absolute ethanol was diluted to the concentrations of 20 μM, 10 μM, 5 μM, and 2 μM. The fluorescence signals were recorded for Phenylalanine (Phe: λ_ex_ 240 nm and λ_em_ 282 nm) (Lakowicz, 2006) and Fmoc-Ala (λ_ex_ 397 nm and λ_em_ 280 nm) (Pappas et al., 2014) and plotted against their respective sample concentrations. The resulting data were subjected to linear regression analysis, which provided the slope and the R-square (COD) for the chromophores of each peptide.

### 4.4 Fourier Transformation Infrared (FT-IR) spectroscopy

IR spectra were measured within the wavenumber range of 4,000 to 650 cm^-1^ using a Cary 630 spectrometer by Agilent. Background and sample spectra were acquired before each acquisition at a spectral resolution of 1 cm^-1^, with 128 scans per measurement.

## 5 Cellular studies

### 5.1 Cell culture and chemicals

BJ, HeLa S3, SW480, and Ht-29 were purchased from ATCC (Manassas, United States of America), and HCT-116 was purchased from Ubigene (Austin, United States of America). MCF10A and 293 Ampho-Phoenix cells were previously described and characterized (Di Giorgio et al, 2021). Cancer cells were cultured in 10% FBS DMEM (Euroclone, Milan, Italy). BJ cells were grown in EMEM (Euroclone, Milan, Italy). Media were supplemented with 10% fetal bovine serum (FBS), L-glutamine (2 mM), penicillin (100 U ml^−1^), and streptomycin (100 μg ml^−1^) (Lonza, Basel, Switzerland). MCF10A was cultured in Ham’s F12: Dulbecco’s modified Eagle’s medium (DMEM) 1:1 medium (Merck, Milan, Italy) supplemented with 5% horse serum (Gibco), penicillin (100 U/ml), streptomycin (100 μg/ml), L-glutamine (2 mM) (Lonza), insulin (0.01 mg/ml), hydrocortisone (500 ng/ml), cholera toxin (100 ng/ml) (Sigma-Aldrich, Milan, Italy), and epithelial growth factor (20 ng/ml) (Peprotech, United Kingdom). Oxaliplatin was purchased from Tocris (Bristol, United Kingdom) and dissolved in DMSO (Sigma-Aldrich, Darmstadt, Germany).

### 5.2 Viability assay and Apo-ONE assay

For the resazurin-to-resorufin reduction assays, cells were grown in a 96-well plate for 120 min at 37 °C with resazurin solution (0.15 mg/ml) (Sigma-Aldrich, Milan, Italy). The fluorescence of resorufin was quantified on a PerkinElmer EnSpire 2,300 Multilabel Reader (ex 550 nm/em 590 nm). For the Apo-ONE assay, 25,000 cells were seeded in a 96-well white polystyrene microplate (Corning, United States of America). After 24 h, cells were treated for 36 h with oxaliplatin and oxaliplatin+SS35. Cells were then lysed and subjected to caspase-3/7 (DEVDase) activity assays by using the Apo-ONE Homogeneous Caspase-3/7 Assay (Promega, Madison, United States of America) according to the manufacturer’s instructions. After 30 min of incubation, fluorescence was recorded in the linear phase of the kinetics with a Synergy H1 plate reader (BioTek, Winooski, United States of America). For the trypan blue viability assay, cells were harvested, pelleted at 1500 rcf, and incubated for 5 min with a PBS/0.4% Trypan Blue solution (Gibco). At least 300 cells were counted for each condition.

### 5.3 Colony formation assay

A total of 25,000 HeLa S-3 cells were seeded in a 96-well plate (Sarstedt, Nümbrecht, Germany). After 24 h, they were treated with 20 µM oxaliplatin or **SS35** (0.1 μg/ml), or both, for 48 h. Then, living cells were harvested and counted. A total of 1000 living cells were seeded in 60-mm adhesion plates (Sarstedt, Nümbrecht, Germany), cultured for 14 days, and stained for 20 min with methylene blue (5 g/L of 50% (v/v) ethanol/water, 1 mL/plate). Colonies were counted with clone.JAR

### 5.4 Cytofluorimetric evaluation of drug uptake

Ht-29 and HeLa S-3 cells were treated with scaling doses of **SS7**, **SS35**, **SS28**, and **SS36** for 3 h; cells were then harvested and resuspended in phosphate-buffered saline (PBS). Single-cell suspensions were prepared as previously described (Di Giorgio et al, 2021), and forward scatter (FSC), side scatter (SSC), and FL-3 were acquired on a BD FACSCalibur cytometer. Data were analyzed with FlowJO software.

### 5.5 Immunoblotting

Cell lysates were obtained by collecting living and dead cells after centrifugation at 1500 rcf for 5 min. Cells were lysed with Laemmli sample buffer 2x. After SDS-PAGE and immunoblotting on nitrocellulose (Whatman, United Kingdom), membranes were incubated overnight with the following primary antibodies: anti-caspase 3 (1:1000 Abcam, ab214430), anti-HA (HA-7, Sigma-Aldrich), anti-actin (MAB1501, Sigma-Aldrich), and anti-histone H3 (D1H2, Cell Signaling Technology). HRP-conjugated secondary antibodies were obtained from Cell Signaling Technology (Danvers, United States of America), and blots were developed with Super Signal West Dura (Pierce, United States of America). For fluorescence-based detection, AF660 or AF760 secondary antibodies were used (Merck, Milan, Italy), and images were acquired at Odyssey M Imaging System (LI-COR Biosciences, United States of America).

### 5.6 Plasmid construction, transfection, retroviral infection, and silencing

pWZL-Hygro Hemagglutinine-XIAP (HA-XIAP) was obtained by subcloning through a restriction-ligation-based approach the ORF of XIAP (BamHI/EcoRI) from pEBB-XIAP (Addgene Plasmid #11558) in pWZL-Hygro HA. Transfections of 293 Ampho-Phoenix cells were carried out with polyethylenimine (PEI, 1 μg/ml) using a 2:1 ratio of PEI (µl): DNA (µg). A pWZL-Hygro empty plasmid was used as a control. Retroviral infection of HeLa S-3 cells was performed with an M.O.I of 0.1–0.3 at 32°C by using Ampho cells as packaging cells. Two consecutive infections were performed, the second 48 h after the first. Cells were selected with 250 μg/ml hygromycin B (Euroclone, Milan, Italy). Polyclonal cultures were then obtained and started to be used 2 weeks after the end of selection. For Integrin Subunit Beta 3 (ITGB3) silencing, 300,000 HeLa cells were seeded in 35-mm plates and transfected with 40 ng endoribonuclease-prepared short interfering RNAs (esiRNAs) (EHU051661, Merck, Milan, Italy) and 5.5 µl Lipofectamine 3,000 (Life Technologies, Carlsbad, United States).

### 5.7 RNA extraction and quantitative qRT-PCR

HeLa cells were lysed using TRIzol (Invitrogen, United States of America). Totally 1.0 μg of total RNA was DNAse I-treated (Ambion, United States of America) and retro-transcribed by using 100 units of M-MLV reverse transcriptase (Life Technologies, United States of America) in the presence of 1.6 μM oligo (dT) and 4 μM random hexamers (Euroclone, Milan, Italy). Real-time quantitative reverse transcriptions (qRT-PCRs) were performed using SYBR Green technology (KAPA Biosystems). Data were analyzed by the comparative threshold cycle (delta delta Ct ΔΔCt) using HPRT and ACTB as normalizers. The primers used for ITGB3 amplification in qRT-PCR are FW: GTG​ACC​TGA​AGG​AGA​ATC​TGC and RV: CCG​GAG​TGC​AAT​CCT​CTG​G.

### 5.8 Statistical analysis

For experimental data, Student’s t-test was employed. The Mann–Whitney test was applied when normality could not be assumed. For comparisons between more than two samples, the ANOVA test was applied coupled with Kruskal–Wallis and Dunn’s multiple comparison tests. Excel and GraphPad Prism were used for statistical analysis. We denoted significance as *p < 0.05, **p < 0.01, ***p < 0.001, and ****p < 0.0001. Unless otherwise indicated, all the data in the figures were represented as arithmetic mean ± the standard deviation from at least three independent experiments.

## 6 Results

### 6.1 Synthesis of peptides

**FIGURE 2:**
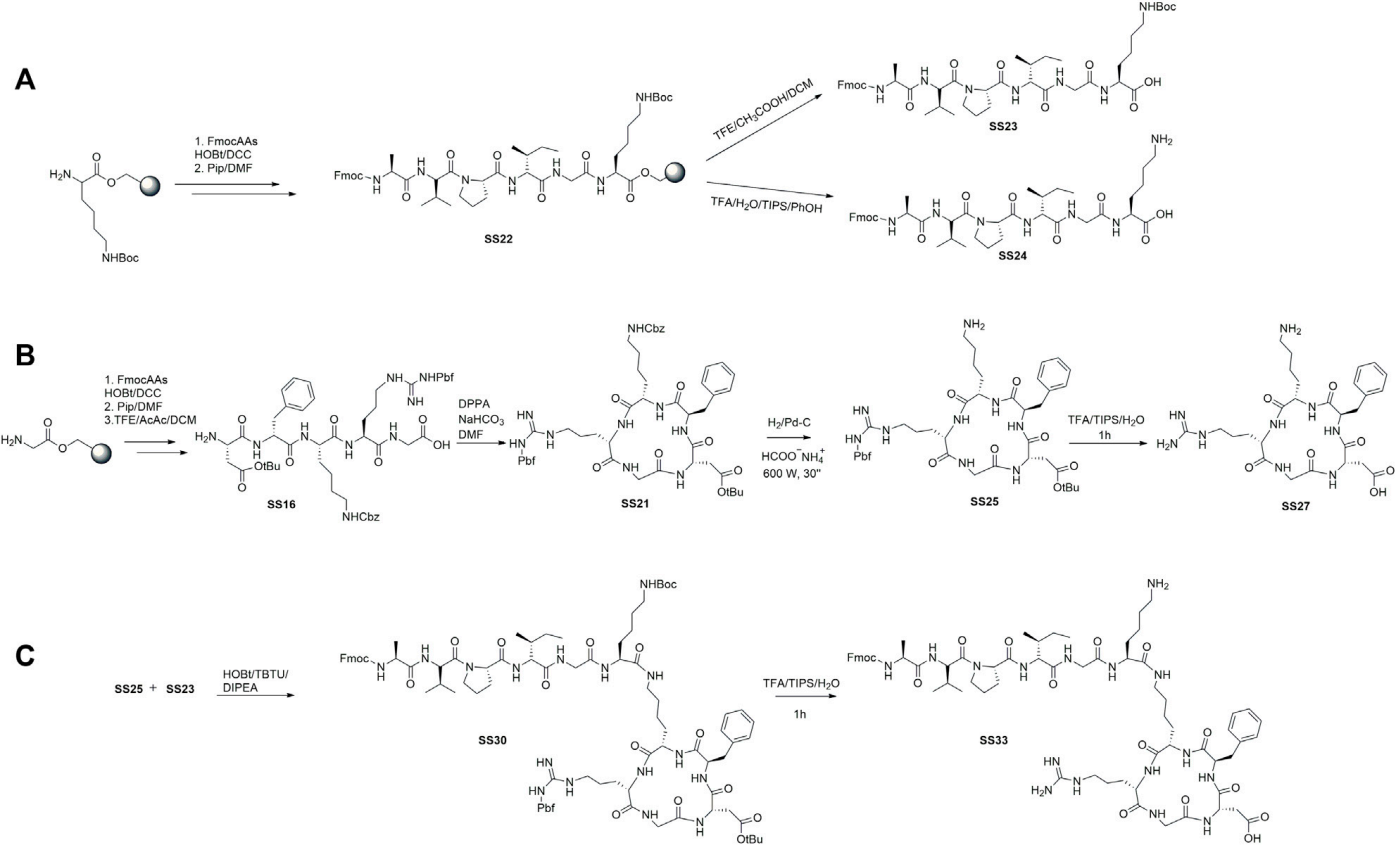
Synthesis of the cytotoxic peptide AVPI **(A)**, integrin ligand αvβ3 c [RGDfK] **(B),** and dual-targeting peptide (DTP) AVPI@c [RGDfK] **(C)**.

To prepare the linear peptides **SS23**, **SS24**, and **SS16,** we used a Cl-Trt resin in the presence of HOBt/DCC under the previously optimized conditions (De Marco et al., 2020). The steps of protective group removal and coupling were repeated until the linear peptides were obtained and recovered by cleavage. **SS24** was cleaved with TFA and scavengers (Figure 1A). For peptides **SS23** and **SS16,** we used TFE and acetic acid in dichloromethane to maintain the protective group in the side chain (Figures 2A,B). The peptides are recognized by analytical RP-HPLC (*see general method*). The cyclization of **SS16** was performed under high-dilution conditions in the presence of DPPA/NaHCO_3_/DMF, yielding c [Arg (pbf)-Gly-Asp(OtBu)-phe-Lys (Cbz)] (Figure 2B). After hydrogenation (Daga et al. 2001) and treatment with TFA, we obtain the cyclic [Arg-Gly-Asp-phe-Lys]. To prepare the dual peptide Fmoc-AVPI@c [RGDfK], the peptides **SS23** and **SS25** were coupled in the presence of HOBt/TBTU/DIPEA. The deprotection under acid conditions with TFA led to obtaining the final product **SS33** (Figure 2C). The peptides were purified by flash chromatography (see *General Procedures*) and characterized by reversed-phase HPLC and 1H NMR. The purities were determined to be >95% by analytical RP-HPLC (see *General Procedures*).

### 6.2 Synthesis of nanoparticles and their functionalization with peptides

**FIGURE 3:**
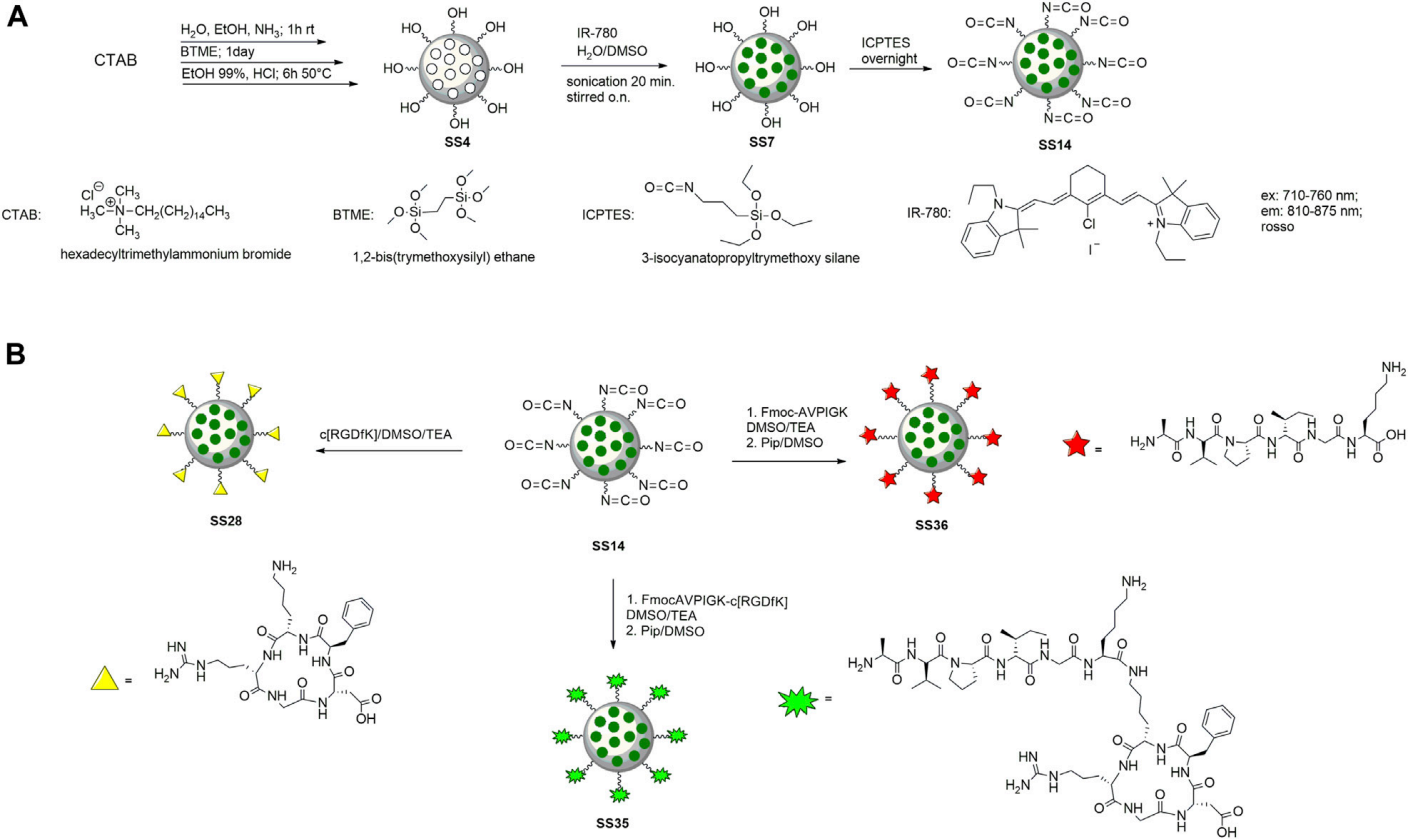
Synthesis of PMO **(A)** and functionalization with cytotoxic peptide (on the left), c [RGDfK] (on the right), and dual-targeting peptide (in the center) **(B)**.

To prepare the nanoparticles, the surfactant CTAB was used to form the micellar solution in the presence of ammonia. BTME was used to form the walls of the nanoparticles, and the excess surfactant was removed by an acid solution of ethanol and HCl (Motealleh et al., 2019). The internal core was loaded with IR-780 according to the literature (Ma et al., 2020) (Figure 3A). Briefly, the dye was dissolved in DMSO and then added to the nanoparticles dispersed in H_2_O. The reaction was stirred for 12 h in the dark, then washed with water, and recovered by centrifugation. To functionalize PMO with peptides, the outer shell of the nanoparticle was reacted previously with ICPTES and TEA, with a procedure optimized in our laboratory (Greco et al., 2015), and then with peptides in basic conditions (Figures 3A,B). The last step is the deprotection of the Fmoc protective group from peptides@PMO to obtain **SS35** and **SS36** with an amine group free of the alanine, which is the pharmacophore responsible for the inhibition of Xiap (Bourguet et al., 2014). PMOs were characterized by DLS, SEM, and IR to analyze the size, shape, and structure after each step.

## 7 Characterization studies

### 7.1 Average particle size (DLS) and scanning electron microscopy (SEM)

**FIGURE 4:**
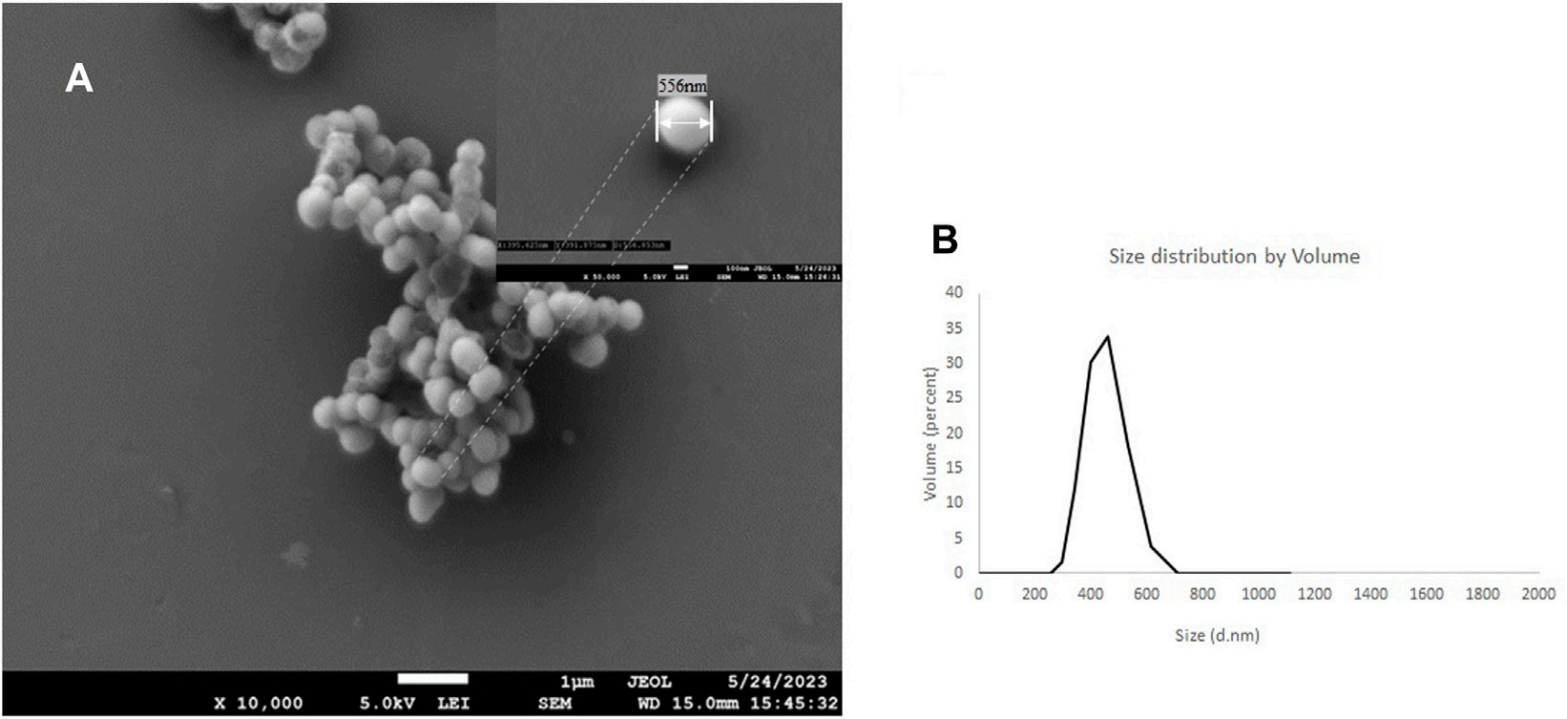
SEM images of the PMO-OH at 10 k of magnification and the inset at 50 k of magnification **(A)** and hydrodynamic distribution by volume determined by DLS (water, 20°C) **(B)**

Scanning electron microscopy shows a PMO-OH having a diameter of 556 nm measured at 50 k magnification, while the hydrodynamic light scattering distribution (dH) measured has a diameter of 495 nm with a PDI of 0.12 (Figure 4). After conjugation with cytotoxic AVPI, cyclicRGD and DTP with ICPTES, which was used to functionalize the outer shell of the nanoparticles, the reactions gave AVPI@PMO, c [RGDfK]@PMO, and DTP@PMO. The increase in size distribution and diameter measured by DLS and SEM confirms the successful functionalization, as the dH after conjugation increased to 856 nm compared with that of native nanoparticles (*see* Supplementary Table S1 and Supplementary Figures S1–S3).

### 7.2 FT-IR

The FT-IR was used to analyze the structure of PMOs. The stretching vibrations at around 693.28 cm^-1^ were assigned to Si-C. The presence of a broad absorption band at 3384.42 cm^-1^ was assigned to Si-OH. The presence of a urea bond is confirmed by stretching vibrations at 1647.48 and at 1546.84 cm^-1^ in **SS28**, at 1651.21 and at 1558.02 cm^-1^ in **SS35**, and at 1654.93 and at 1558.02 cm^-1^ in **SS36** spectrums (*see* Supplementary Material S4–S8) between the amino group of the peptides and PMOs.

### 7.3 Determination of PMO peptide functionalization

Once calibration curves were established, solutions of **SS28**, **SS35,** and **SS36** (ca.1 mg) in ethanol (2 mL) were prepared, and the absorption spectra were measured. The absorbance values of peptides linked to PMOs were adjusted to account for potential interference caused by PMOs, which can scatter or absorb light in the visible and UV ranges, leading to inaccuracies in absorbance measurements of chromophores. The absorbance spectrum of PMO was subtracted from the absorbance spectrum of the sample containing the peptide@PMO. In the region of absorption of the chromophore, we observed a linear and well-defined relationship between the absorbance of PMO and wavelength. To generate the PMO absorbance spectrum under the chromophore’s absorption band, we performed linear regression on the absorbance values outside the chromophore’s band. This allowed us to establish a baseline for the absorption of the PMO. The amount of peptide functionalized on the PMO was determined using the formula obtained from the calibration curves. The absorbance is at λ_max_ = 257 nm for Phe and λ_max_ = 267 nm for Fmoc-Ala. The quantification values are reported in Table 1 (for calibration curves, see Supplementary Material S9, S10).

**TABLE 1:** Estimated number of peptides conjugated to each NP via urea binding determined by absorbance measurements using Phe and FmocAla as standards to construct the calibration curves. * Peptide amount binding for each milligram of PMO.

Peptide	Peptide/PMO (mg)*
FmocAVPI@PMO	0.074
c [RGDfK]@PMO	0.42
DTP@PMO	0.16

## 8 Biological anti-cancer effects

### 8.1 Uptake

**FIGURE 5:**
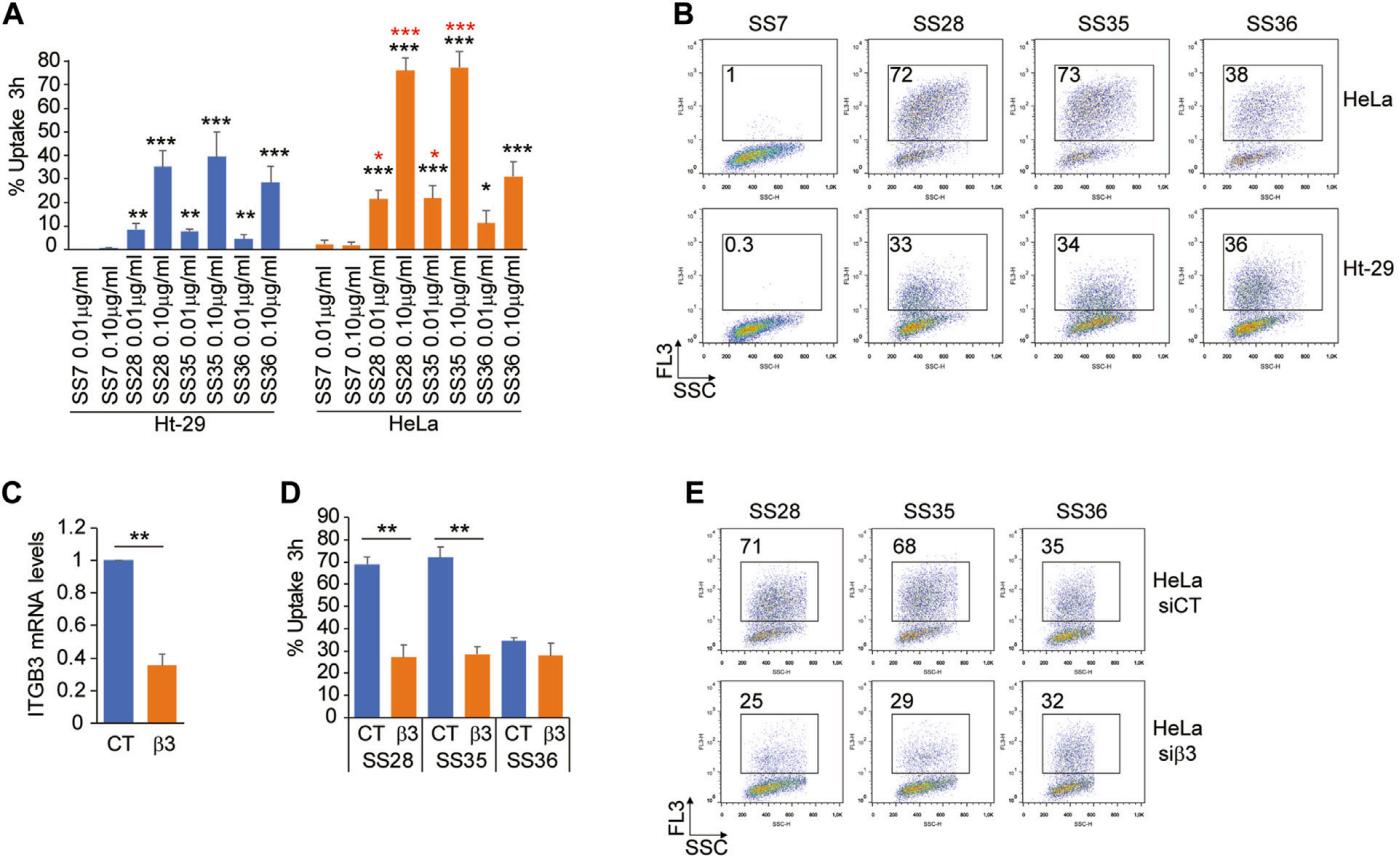
Integrin αvβ3 binding promotes the uptake of **SS28- and SS35** nanoparticle-based compounds. **(A)** Ht-29 and HeLa S-3 cells were treated for 3 h with the indicated concentrations of **SS7**, **SS28**, **SS35,** and **SS36**. Then, the cells were harvested and subjected to cytofluorimetric analysis to identify IR-780-positive cells. Data are represented as mean ± standard deviation, n = 4. **(B)** Representative dot plots obtained from the indicated cells treated as in Figure 5A. In each panel, the percentage of FL-3 (IR-780)-positive cells is indicated. **(C)** mRNA relative expression level of ITGB3 in HeLa S-3 cells transfected for 48 h with esiCT or esiRNA directed against human ITGB3. Data are represented as mean ± standard deviation, n = 3. **(D)** HeLa S-3 cells were silenced for 48 h, as explained in Figure 5C, and then were treated with 0.1 μg/ml **SS28**, **SS35,** and **SS36**. After 3 h, the cells were harvested and subjected to cytofluorimetric analysis to identify IR-780-positive cells. Data are represented as mean ± standard deviation, n = 3. **(E)** Representative dot plots obtained from the indicated cells treated as in Figure 5D. In each panel, the percentage of FL-3 (IR-780)-positive cells is indicated.

To prove the successful uptake of the three molecules (**SS28, SS35,** and **SS36**), two cancer cell lines were selected: Ht-29, a human colorectal adenocarcinoma cell line expressing low levels of the heterodimeric αvβ3 integrin, and HeLa, a human cervical epithelioid carcinoma cell line expressing high amounts of αvβ3 (De Marco et al., 2020). Equal amounts of Ht-29 and HeLa cells, which were in the exponential growth phase, were loaded with 0.01 μg/ml and 0.1 μg/ml of the three functionalized nanoparticles **SS28**, **SS35,** and **SS36** for 3 hours. Cellular uptake was monitored by flow cytometry by detecting the fluorescence of IR-780 loaded into the internal core of the nanoparticles. Background autofluorescence was quantified by treating the cells with empty, non-fluorescent nanoparticles **SS4**. As shown in Figure 5A, the uptake of **SS36** (nanoparticles functionalized with AVPI) was equivalent between Ht-29 and HeLa cells. A more efficient uptake by HeLa cells was observed for **SS28** (nanoparticles functionalized with RGD peptide) and **SS35** (nanoparticles functionalized with RGD and AVPI) (Figure 5A, B). This evidence supports the efficacy of RGD-conjugates as targeting ligands for αvβ3 positive cancer cells, as previously shown by others (Danhier *et al., 2012*).

To prove that the differential uptake of **SS28** and **SS35** between Ht-29 and HeLa cells was indeed due to differential expression levels of αvβ3, we silenced ITGB3 expression in HeLa cells for 48 h and monitored the uptake of **SS28**, **SS35,** and **SS36** for 3 hours after administration of the compounds. In HeLa cells successfully deprived of the integrin β3 (Figure 5C), the uptake of **SS28** and **SS35**, but not of **SS36**, was severely impaired and comparable to the uptake of **SS36** (Figure 5D, E). These data indicate that integrin β3 binding promotes the uptake of **SS28-** and **SS35** nanoparticle-based compounds.

### 8.2 **SS35** induces apoptosis by inhibiting IAPs in αvβ3-positive cancer cells

**FIGURE 6:**
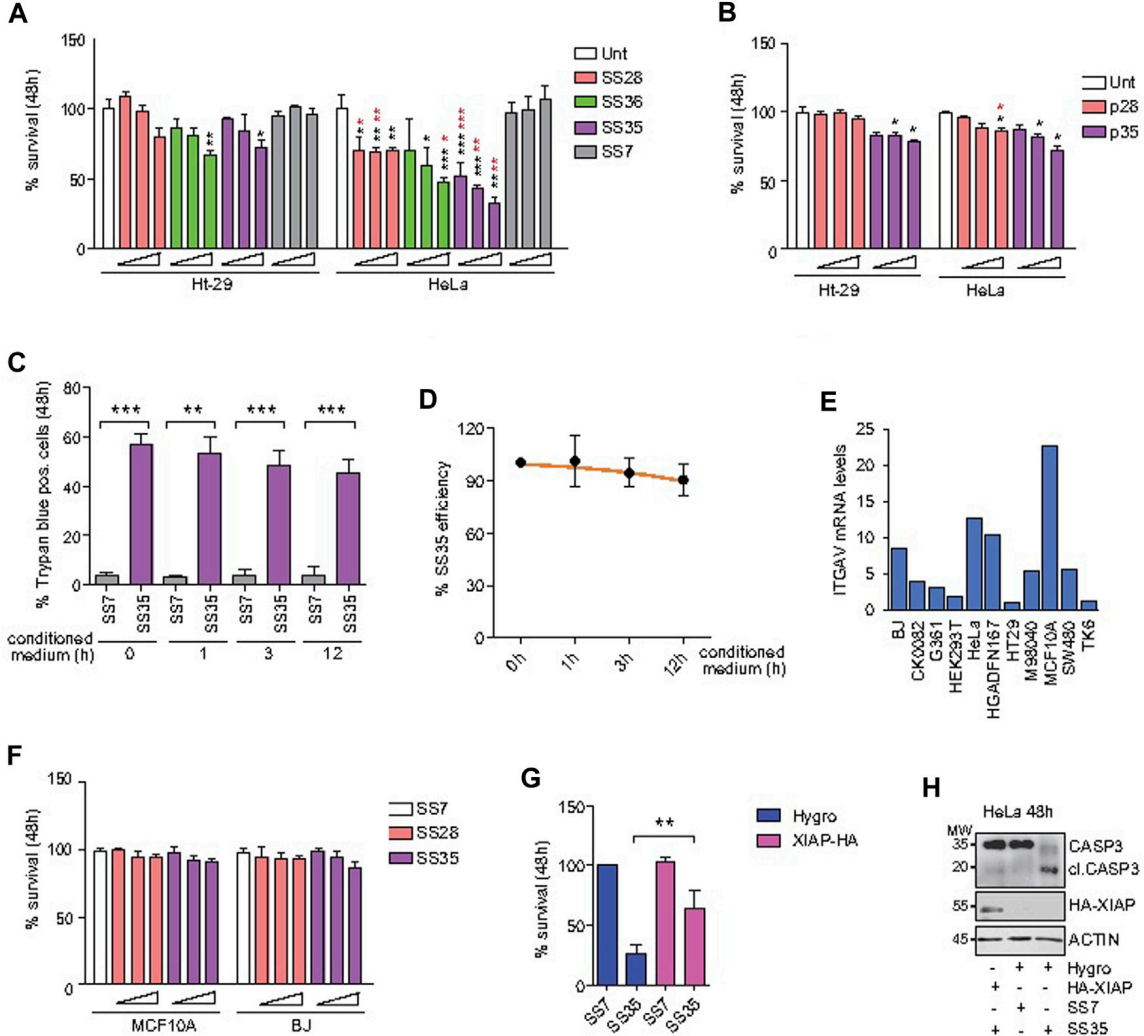
**SS35** induces apoptosis by inhibiting IAPs in αvβ3-positive cancer cells, but not in normal healthy cells. **(A)** Histogram representing the percentage of viable cells actively metabolizing resazurin after 48 h of treatment with increasing doses (0.02, 0.05, and 0.10 μg/ml) of the indicated compounds. Data are represented as mean ± standard deviation, n = 5. The significance with respect to untreated cells is marked with black asterisks; the significance obtained by comparing each treatment efficacy between HeLa S-3 (αvβ3 positive) and Ht-29 cells (αvβ3 negative) is marked with red asterisks. **(B)** Histogram representing the percentage of HeLa and Ht-29 viable cells actively metabolizing resazurin after 48 h of treatment with 1, 5, and 25 µM of the RGD free-peptide (named p28 in analogy to the nanoparticle-conjugated peptide **SS28**) and RGD-AVPI chimeric peptide (named p35 in analogy to the nanoparticle-conjugated peptide **SS35**). Data are represented as mean ± standard deviation, n = 3. **(C)** Histogram representing the percentage of trypan blue-positive HeLa cells treated for 48 h with 0.10 μg/ml **SS7** or **SS35**, as indicated. In both the cases, the molecules were pre-incubated for the indicated time in the conditioned medium collected from 24 h serum-starved HeLa cells (1*10^6 cells/ml) to expose them to extracellular proteases. **(D)** **SS35** efficiency was calculated for the indicated time points (h) of exposure to the extracellular environment, as explained in Figure 6C, relatively to the unexposed molecule (time 0 h). **(E)** ITGAV mRNA levels in the indicated healthy and cancer cell lines, relatively to Ht-29 considered 1. **(F)** Histogram representing the percentage of viable MCF10A and BJ cells actively metabolizing resazurin after 48 h of treatment with increasing doses (0.02, 0.05, and 0.10 μg/ml) of the indicated compounds. Data are represented as mean ± standard deviation, n = 3. **(G)** Histogram representing the percentage of HeLa S-3 viable cells actively metabolizing resazurin after 48 h of treatment with 0.10 μg/ml **SS7** and **SS35**. HeLa cells stably overexpressing Hygro or HA-XIAP were compared. Data are represented as mean ± standard deviation, n = 3. **(H)** Immunoblot analysis of the indicated HeLa cells treated for 48 h with the indicated compounds. Anti-HA was used to detect HA-XIAP. Actin was used as the loading control. The immunoblot was repeated three times with similar results.

After establishing the differential uptake of the described compounds in Ht-29 and HeLa cells, we performed standard biological assays to evaluate the induction of apoptosis. First, Ht-29 and HeLa cells were treated with increasing doses (0.02, 0.04, and 0.06 μg/ml) of **SS28**, **SS35**, and **SS36** for 48 h, and cell viability was assessed by resazurin reduction assays (Figure 6A). HeLa cells were found to be more sensitive to **SS35** than to Ht-29 (Figure 6A), which is in excellent agreement with the uptake data (Figures 5A, B). Both Ht-29 and HeLa cells were moderately sensitive to **SS36**, whereas **SS28** resulted in some toxicity only in HeLa cells (Figure 6A). Interestingly, increasing doses of **SS28** did not correlate with an increase in toxicity, suggesting that a) the engagement of αvβ3 decreases HeLa fitness and b) the lower dose is sufficient to block αvβ3 and achieve efficacy.

To evaluate the superior efficacy of our peptide-to-nanoparticle conjugation system, we decided to evaluate the cytotoxicity of the free peptides. For this purpose, Ht-29 and HeLa cells were treated with 1, 5, and 25 µM of the free RGD peptide (referred to as p28 by analogy with the nanoparticle-conjugated peptide **SS28**) and the chimeric peptide RGD-AVPI (referred to as p35 by analogy with the nanoparticle-conjugated peptide **SS35**) (Figure 6B). Although the two experiments cannot be directly compared as the conjugation to the nanoparticles may affect other aspects than the bioavailability of the peptides, like their stability and activity, by comparing Figures 6A,B, it is clear that the effect of the unconjugated molecules is significantly attenuated. One of the reasons for the higher functionality of the peptides conjugated with nanoparticles compared to the unconjugated peptides could be their higher stability than that of the naked peptides, in addition to their higher uptake, as reported by others and reviewed in Lucana et al (2021). To indirectly test the stability of the nanoparticle-conjugated **SS35** peptide, we exposed the **SS35** molecule, and the empty nanoparticle **SS7** as a control, to the action of extracellular proteases for 0, 1, 3, and 12 h, and then we tested their residual activity by evaluating their cytotoxicity on HeLa cells. **SS35** was found to retain significant activity even after 12 h in an extracellular environment at 37°C (Figures 6C,D).

In molecular oncology, apoptosis inducers must not only be functional but also have selectivity toward cancer cells. As the expression level of αv integrin is heterogeneous in healthy tissues, we selected MCF10A and BJ to test the cytotoxicity of **SS28** and **SS35**, as these normal cell lines were characterized by the highest mRNA levels of *ITGAV* on the basis of what emerges from the analysis of the transcriptome available on GENT2 (Park et al, 2019) (Figure 6E). MCF10A and BJ cells were loaded with increasing doses (0.02, 0.05, and 0.10 μg/ml) of **SS28** and **SS35**, and no significant alteration of their viability was observed after 48 h of treatment (Figure 6F).

Finally, to prove that the high cytotoxicity of **SS35** was due to the inhibition of IAP through AVPI, a retroviral plasmid was used to stably overexpress HA-XIAP in HeLa cells (Figure 6G). A retroviral empty plasmid encoding for hygromycin B resistance was used as a control. Polyclonal HeLa cultures overexpressing HA-XIAP and hygromycin B resistance or hygromycin B resistance alone, as a control, were established. As shown in Figure 6G, HA-XIAP overexpressing cells were much less sensitive to **SS35** than hygro-expressing control cells (Figure 6G). This was correlated to a defective cleavage of caspase 3 in HA-XIAP overexpressing cells (Figure 6H).

Overall, these data demonstrate that the selective targeting of αvβ3-positive cells with the **SS35** molecule leads to the induction of apoptosis, which can be overcome by increasing XIAP levels. Importantly, **SS35** does not negatively impact the viability of the healthy cells evaluated, thus confirming its potential specific anti-neoplastic effect.

### 8.3 **SS35** potentiates the cytotoxic effects of oxaliplatin in cancer cells

**FIGURE 7:**
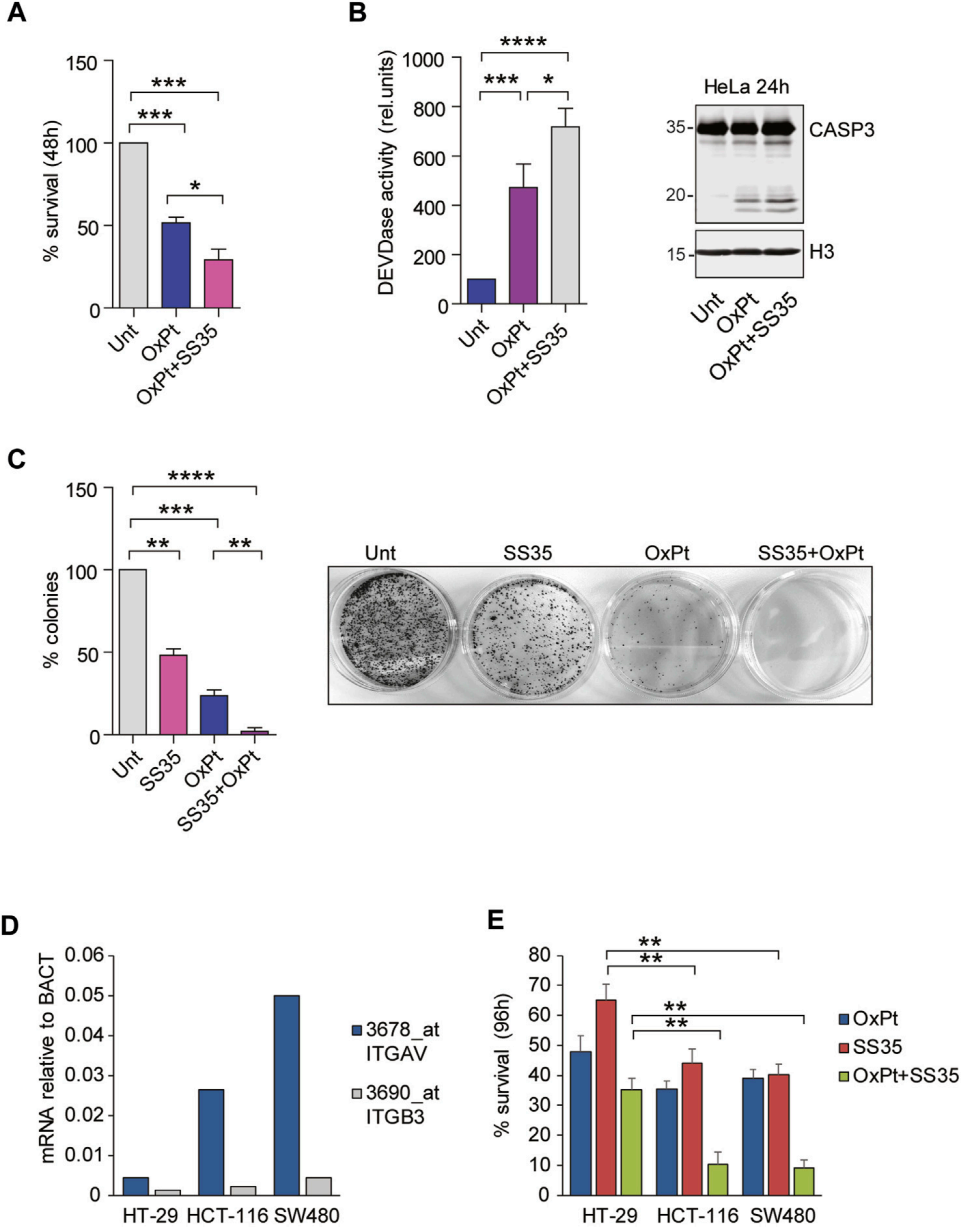
**SS35** potentiates the cytotoxic effects of oxaliplatin in αvβ3-positive cancer cells. **(A)** Histogram representing the percentage of viable HeLa cells actively metabolizing resazurin after 48 h of treatment with 20 µM oxaliplatin and 0.10 μg/ml **SS35**, as indicated. Data are represented as mean ± standard deviation, n = 3. **(B)** Caspase activation (DEVDase activity) in HeLa cells, treated for 36 h, as described in Figure 7A. Data are expressed as mean ± S. D, n = 3. **(C)** Colony formation assay in HeLa cells treated for 48 h with 20 µM oxaliplatin or **SS35** (0.1 μg/ml) or both. A total of 1000 living cells were seeded in 60-mm adhesion plates and cultured for 14 days. Data are represented as mean ± standard deviation, n = 3. On the right, a representative image of methylene blue stained colonies is shown. **(D)** mRNA expression levels of ITGAV and ITGB3 in the indicated colorectal cancer cell lines with respect to beta actin. Data were obtained by re-analyzing the GSE36133 dataset. **(E)** Histogram representing the percentage of the indicated viable colorectal cancer cells actively metabolizing resazurin after 96 h of treatment with 20 µM oxaliplatin and 0.10 μg/ml **SS35**, as indicated. Data are represented as mean ± standard deviation, n = 3.

Inherent or acquired resistance to platinum compounds negatively affects their efficacy against cervical cancer (Bhattacharjee et al., 2022). Overexpression of anti-apoptotic Bcl2 family members is one of the most common resistance mechanisms. We therefore hypothesized that treatment with **SS35** would enhance the toxicity of oxaliplatin (OxPt). To prove this, we treated HeLa cells with 10 µM OxPt as a single agent or in combination with 0.6 μg/ml **SS35**. As shown in Figure 7A, **SS35** increases the anti-cancer activity of OxPt, and this effect correlates with increased activity of caspases 3 and 7 (Figure 7B) and increased proteolysis of caspase 3 (Figure 7B). Importantly, **SS35** and OxPt showed synergistic anti-cancer effects in a 14-day colony formation assay, achieving a nearly complete response (Figure 7C). These data suggest that **SS35** sensitizes αvβ3-positive HeLa cells to the genotoxic compound oxaliplatin.

To demonstrate that the cytotoxic effect of **SS35** was not restricted to HeLa cells but can be expanded to other αvβ3-positive cancer cells, we adopted a panel of three colorectal cancer (CRC) cell lines, Ht-29, HCT-116, and SW480, that were previously described for having increasing expression levels of αvβ3: Ht-29 << HCT-116 < SW480 (Chin et al. 2019; Lei et al., 2011; Figure 7D). FOLFOX (folinic acid, 5-fluorouracil, and oxaliplatin) is the most commonly used neoadjuvant and adjuvant chemotherapy regimen for advanced colorectal cancers (CRC), and resistance to OxPt was observed in non-responsive patients (Hoang et al. 2022). Targeting αvβ integrins proved to be effective in potentiating OxPt effects in CRC cells (Dia and de Meija 2011). Engagement of αvβ3 was successfully exploited as a safe and precise exosome-based-therapy targeting strategy in CRC murine models to overcome resistance (Lin et al., 2021). For these reasons, we treated our selected panel of CRC cells with 6 µM OxPt and 0.6 μg/ml **SS35**, separately and in combination. Resazurin reduction assays were performed 96 h after the treatment to quantify cell survival (Figure 7E). **SS35** proved to have limited efficacy in potentiating the OxPt effect in Ht-29, while being successful in strongly decreasing the viability of HCT-116 and SW480 cells in combined treatments.

Overall, our data suggest that the adoption of nanoparticles loaded with Smac mimetics and the ability to engage αvβ3 could be a good strategy to enhance the effects of oxaliplatin in αvβ3-positive cancers.

## 9 Discussion and conclusion

In the current study, we synthesized a dual-targeting peptide binding on the nanoparticles, as a mimetic of the cytotoxic protein Smac/DIABLO. DTP is formed by the short sequence AVPI, responsible for the inhibition of the protein IAP and activation of apoptosis in cancer cells (Rathore et al., 2017), and the cyclic penta-peptide RGDfK, an antagonist of the integrin αvβ3, which confers selectivity toward cancer cells (Haubner et al., 1997). Nanoparticles have the role of nanocarriers, which is to improve the bioavailability and biodistribution of peptides in the cancer cells (Dadwal et al., 2018). The cytotoxic peptide was prepared with a lysine as the last amino acid to permit the following steps: 1) coupling with **SS25** to form the DTP and 2) the functionalization on the nanoparticles. To prepare the peptides, we used solid-phase peptide synthesis, as reported in Bourguet et al. (2014) and Hamdan et al. (2019). In summary, the coupling steps were performed in DMF, with HOBt and DCC as coupling reagents at room temperature. The deprotection steps were carried out in piperidine/DMF (ratio 1:3). The coupling and deprotection steps are monitored by a colorimetric test and repeated until the final products are obtained (see the aforementioned procedure). To prepare **SS23** and **SS16,** the cleavage was carried out with TFE and acetic acid to maintain the protective group (Figures 2A,B). For **SS24**, the cleavage was carried out with TFA and scavengers; in these conditions, the protective group was completely removed (Figure 2A). To prepare the dual-targeting peptide **SS33**, the linear cytotoxic peptide (**SS23)** and cyclic RGD (**SS25)** were coupled in standard conditions and then deprotected to yield the final compound **SS33** (Figure 2C). The peptides were functionalized on periodic mesoporous organosilica nanoparticles. These nanoparticles feature tunable pores to allow the loading of large drugs or peptides and a high surface area to allow for chemical functionalization to use them as nanocarriers. PMO was prepared starting from a CTAB, in water and ammonia, to form the micelles and then added BTME to form the wall of the nanoparticles. After 1 day, the excess of CTAB was removed by treatment with an acid solution of ethanol/HCl. A dye was introduced into the pores of the PMO, and the outer shell was functionalized using ICPTES (see Figure 3A and the aforementioned procedure for details) (Guan et al., 2012). To prepare **SS28**, **SS35,** and **SS36**, the peptides were conjugated on the PMO by a urea bond. To maintain the cytotoxic activity in **SS35** and **SS36**, AVPI and DTP reacted in the presence of the Fmoc protective group on the *N*-terminal of alanine. This strategy allowed to selectively bind the peptides to the nanoparticles without involving the amino group of alanine responsible for the cytotoxic activity. Smac has been shown to form an arc-shaped homodimer that allows the N-terminus of alanine to interact with the XIAP–BIR3 complex via van der Waals interactions. The loss of the amino group leads to the loss of interactions with the complex and consequently of the cytotoxic activity of Smac/DIABLO (Wu et al. 2000). Deprotection is carried out after conjugation with PMOs (Figure 3B), and the disappearance of the protective group was confirmed by the Kaiser test.

The size of PMOs was analyzed by DLS and confirmed by SEM, where we observed the sphericity of the nanoparticles (Figure 4). PMO-OH showed a hydrodynamic distribution of 495 nm. After conjugation with DTP, the size was improved at 725 nm, compared to that of the primeval PMO-OH. The presence of a protective group on the N-terminus of alanine in AVPI and in the DTP during the functionalization is important to maintain the pharmacophore. The crystal structures of SMAC show an arc folding rich in hydrophobic van der Waals-type interactions. Alterations in these interactions lead to key residues at the interface disrupting dimer formation and significantly reducing the ability of Smac to activate caspase-3. Furthermore, the N-terminal residues of Smac are essential for Smac function since alteration of the first amino acid renders the resulting protein completely inactive (Chai et al., 2000).

FT-IR analysis showed the presence of characteristic peaks and further confirmed the occurrence of functionalization of the peptides on the nanoparticles. In **SS4**, the presence of stretching vibration at 3384 cm^-1^ shows the presence of Si-OH, and the broad peak at 1088 cm^-1^ corresponds to the SiO stretching vibrations (Zhao et al., 2018). The peak around at 693 cm^-1^ indicates stretching vibrations and has been assigned to Si-C; the C-H deformation vibrations at 2896 cm^-1^, 1412 cm^-1^, and 1271 cm^-1^ are due to the presence of the silsesquioxane structure (Dìaz-Morales et al., 2006). The disappearance of the peak at 3384 cm^-1^ in **SS14** and the appearance of peaks at approximately 2288 cm^-1^ indicate the presence of the isocyanic group (Soares et al., 2022). The stretching vibrations at approximately 1650 cm^-1^ and 1550 cm^-1^ in **SS28**, **SS35,** and **SS36** and the disappearance of the peak of the isocyanic group confirm the formation of the urea bond and the conjugation of peptides on the nanoparticles (Sun and Xue, 2015).

To quantify the amount of peptide loading on the PMOs, we used Fmoc Alanine (λ_ex_ 397 nm and λ_em_ 280 nm) and Phenylalanine (λ_ex_ 240 nm and λ_em_ 282 nm) to establish the calibration curves (Vázquez et al, 2004). We found 0.074 mg of AVPI, 0.42 mg of c [RGDfK], and 0.16 mg of DTP per milligram of PMO (Table 1).

The functionalization of the nanoparticles with RGD peptides to engage αvβ3 integrins proved to be successful in increasing the uptake of **SS28** and **SS35** by αvβ3-positive cancer cells. This is consistent with what was previously reported (Danhier et al 2012; Wang et al. 2013). αvβ3 targeting may raise some concerns as i) not all the cancer cells express αvβ3 and ii) endothelial cells express higher levels of αvβ3 during angiogenesis (Steri et al., 2014). However, RGD-based targeting proved successful in blocking cancer cell migration and invasiveness (Rolli et al., 2003) and the process of neoangiogenesis (Steri et al., 2014).

**SS35** triggers apoptosis in HeLa cells and in all the αvβ3-positive colorectal cancer cells tested. When used in combination with oxaliplatin, **SS35** treatment potentiates the induction of cell death, achieving almost a complete response. Oxaliplatin was selected for the combined treatment as platinum-based compounds are widely used in neoadjuvant and adjuvant therapies for advanced cervical (Lukka et al., 2002; Rosa et al., 2012) and colorectal cancers (Andrè et al., 2004; Huang et al., 2021). Moreover, Smac mimetics were reported to sensitize colorectalcancer cells (Fichtner et al., 2020) and cervical cancer cells (Benetatos et al., 2014) to extrinsic apoptosis (Kroemer et al., 2009), necroptosis (Benetatos et al., 2014), and immunotherapy (Beug et al., 2017). Mechanistically, Smac mimetics have been reported to respond to autocrine TNFα signaling and promote the formation of a RIPK1-dependent caspase-8-activating complex, thus pre-sensitizing cancer cells to pro-apoptotic stimuli (Petersen et al., 2007). As oxaliplatin was reported to trigger extrinsic apoptosis (Yang et al., 2012; Zanardelli et al., 2015), the additive effects obtained in our experiments by co-treating cancer cells with OxPt and **SS35** are not informative about the specific mechanism of cell death triggered. In particular, further experiments are required to clarify whether **SS35** would be effective in sensitizing caspase-8 mutated cells to OxPt.

As it has been reported that inhibition of αvβ3 ligation to the matrix induces anoikis in different cancer cells (Montgomery et al., 1994; Silginer, et al., 2014; Dolinschek, et al., 2020) and blocks cancer migration and invasiveness (Brooks et al., 1996; Kiosses et al., 2001; Liu et al., 2008; Missirlis et al., 2016), it would be worthwhile to better elucidate whether the **SS35** molecule would also perturb integrin engagement, maybe increasing the toxicity of the AVPI peptide. Specific *in vivo* experiments are required to evaluate this possibility, as the engagement of αvβ3 is expected to be transient and reversible. In particular, additional *in vivo* experiments will allow us to evaluate the efficacy of **SS35** in remodeling tumor matrix interactions, as well as its pharmacodynamics and its ability to modulate the tumor microenvironment. However, despite the limitations of the cellular models used, our data demonstrate that RGD-coated nanoparticles provide an efficient way to target tumor cells overexpressing αvβ3 integrin with SMAC mimetics.

In conclusion, we demonstrated the efficacy of DTP@PMO as a mimetic of Smac/DIABLO. **SS35** shows the synergic effect when used with oxaliplatin, which is interesting for the development of new cancer therapies.

## Data Availability

The original contributions presented in the study are included in the article/Supplementary Material; further inquiries can be directed to the corresponding author.
