# Supplementary material for: Dual-targeting peptides@PMO, a mimetic to the pro-apoptotic protein Smac/DIABLO for selective activation of apoptosis in cancer cells
Source: Front Pharmacol. 2023 Aug 29;14:1237478. doi: 10.3389/fphar.2023.1237478 (PMC10497945; doi:10.3389/fphar.2023.1237478)

Supplementary Material

PMO	Particle size* (by volume)/nm	Polydispersity index (PDI)
PMO-OH **(SS4)**	495±131.9	0.12±0.39
AVPI@PMO (**SS36**)	650±395	0.51±0.19
c[RGDfK]@PMO (**SS28**)	900±395	0.45±0.19
DTP@PMO (**SS35**)	725±395	0.82±0.19

*Data expressed as mean ±standard deviation ($n = 3$).

Supplementary Table S1 Summary of hydrodynamic size distribution by volume (20°C) of samples used in this work.

Supplementary Figure S1 SEM images of the AVPI@PMO (**SS36**) at 25k of magnification and inset at 50k of magnification (**A**) and hydrodynamic distribution by volume determined by DLS (water, 20°C) (**B**).

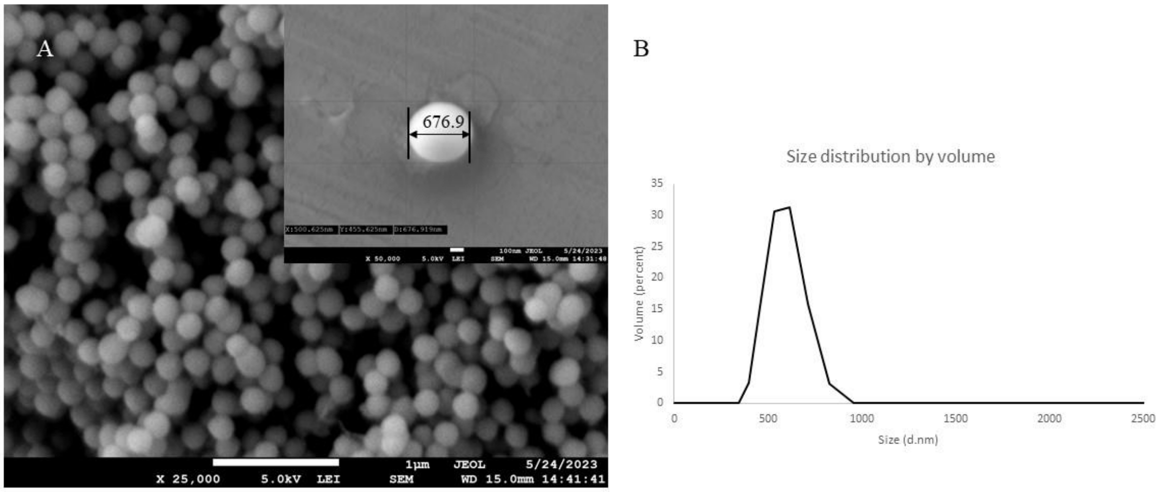

Supplementary Figure S2 SEM images of the c[RGDfK]@PMO (**SS28**) at 10k of magnification and inset at 50k of magnification (**A**) and hydrodynamic distribution by volume determined by DLS (water, 20°C) (**B**)

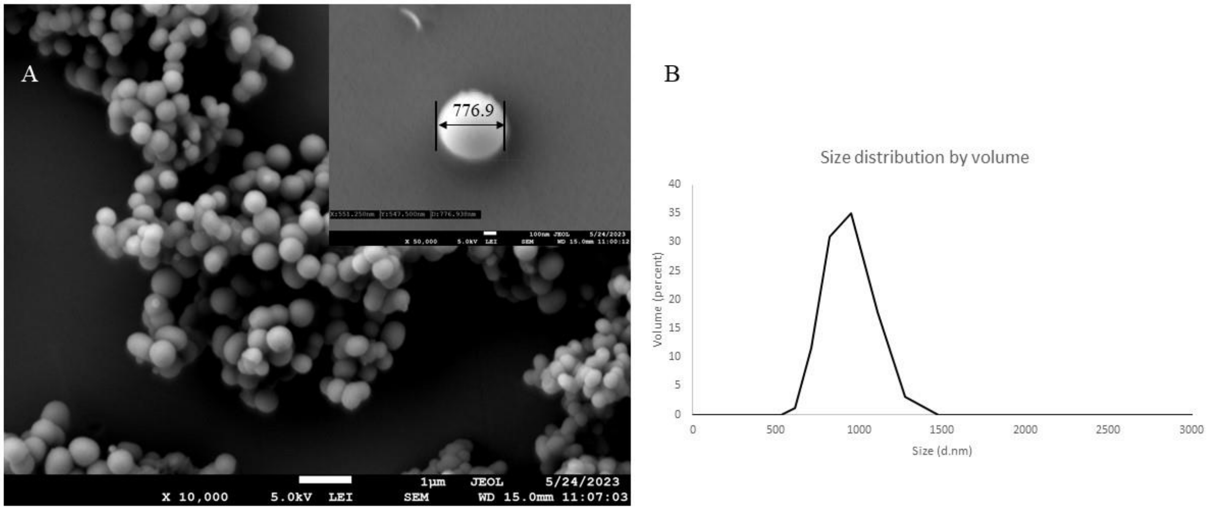

Supplementary Figure S3 SEM images of the AVPI-c[RGDfK]@PMO (DTP@PMO) (**SS35**) at 10k of magnification and inset at 50k of magnification (**A**) and hydrodynamic distribution by volume determined by DLS (water, 20°C) (**B**)

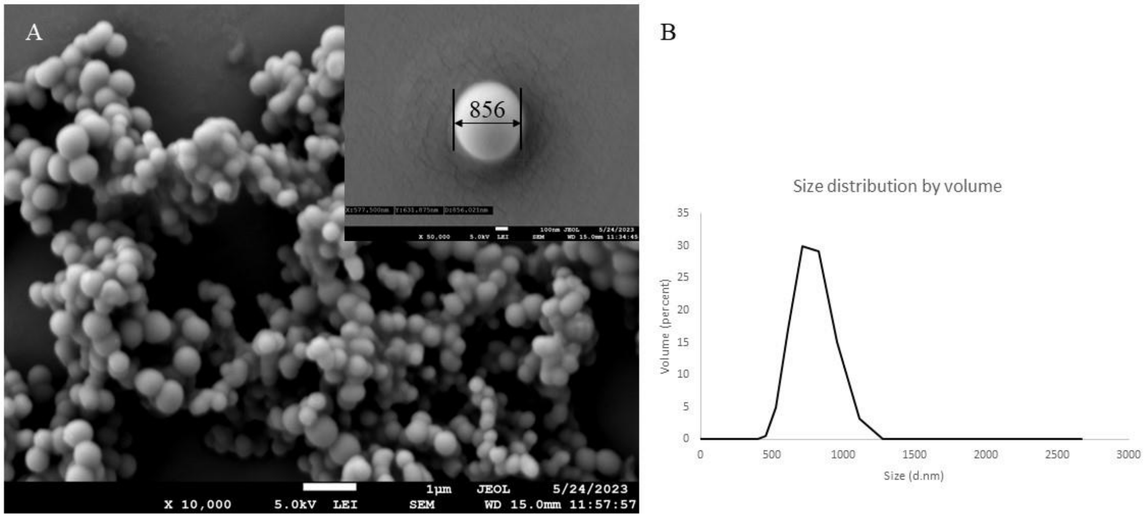

Supplementary Figure S4 IR-spectrum of PMO-OH (**SS4**) were measured within the wavenumber range of 4000 to 650 cm^{-1}.

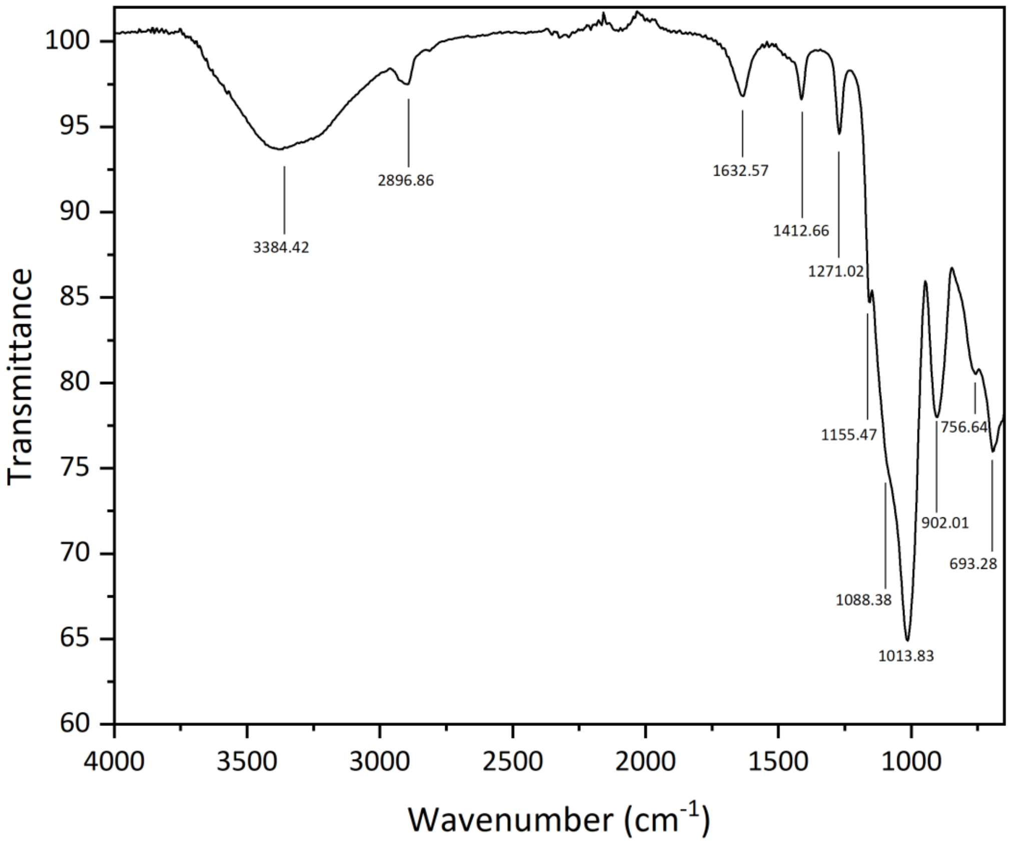

Supplementary Figure S5 IR-spectrum of ICPTES@PMO (**SS14**) were measured within the wavenumber range of 4000 to 650 cm^{-1}.

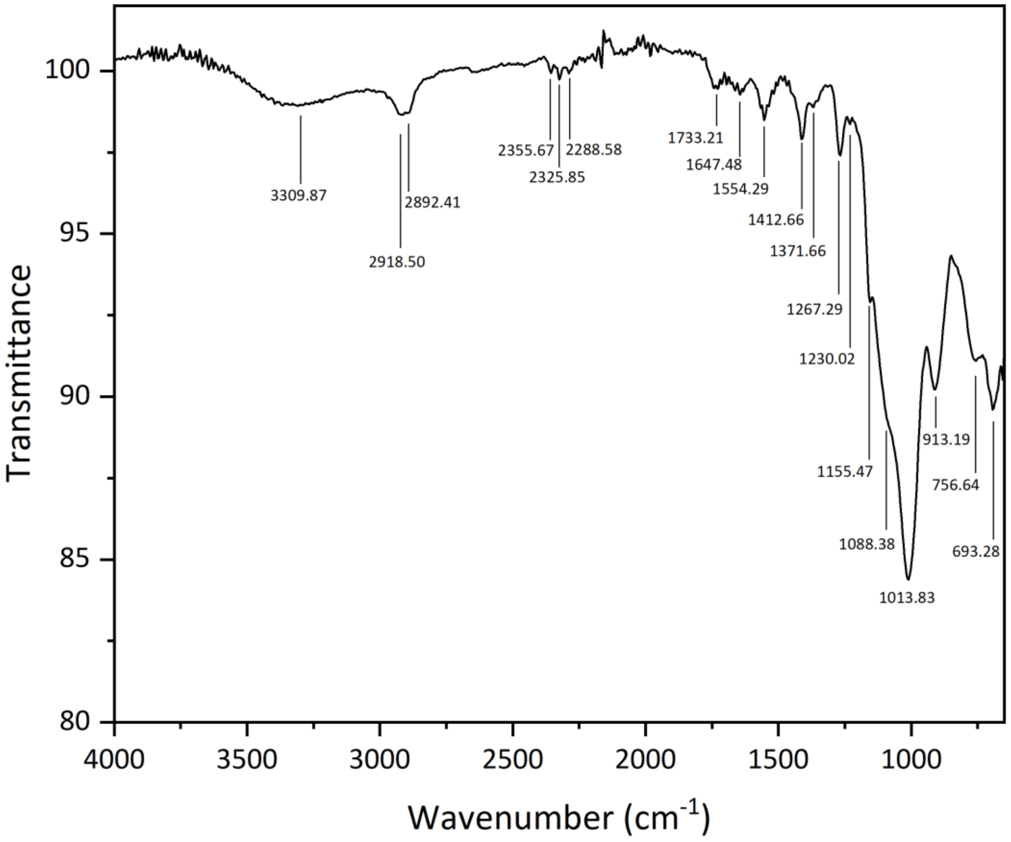

Supplementary Figure S6 IR-spectrum of c[RGDfK]@PMO (**SS28**) were measured within the wavenumber range of 4000 to 650 cm^{-1}.

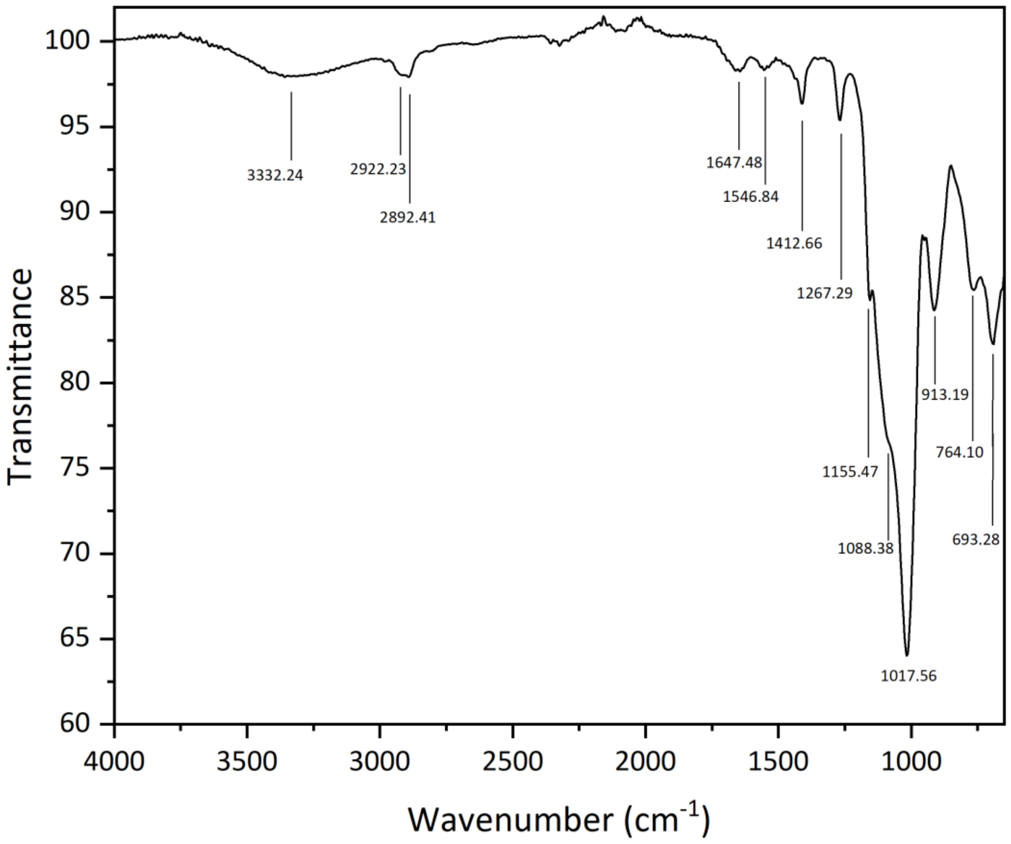

Supplementary Figure S7 IR-spectrum of AVPI-c[RGDfK]@PMO (DTP@PMO) (**SS35**) were measured within the wavenumber range of 4000 to 650 cm^{-1}.

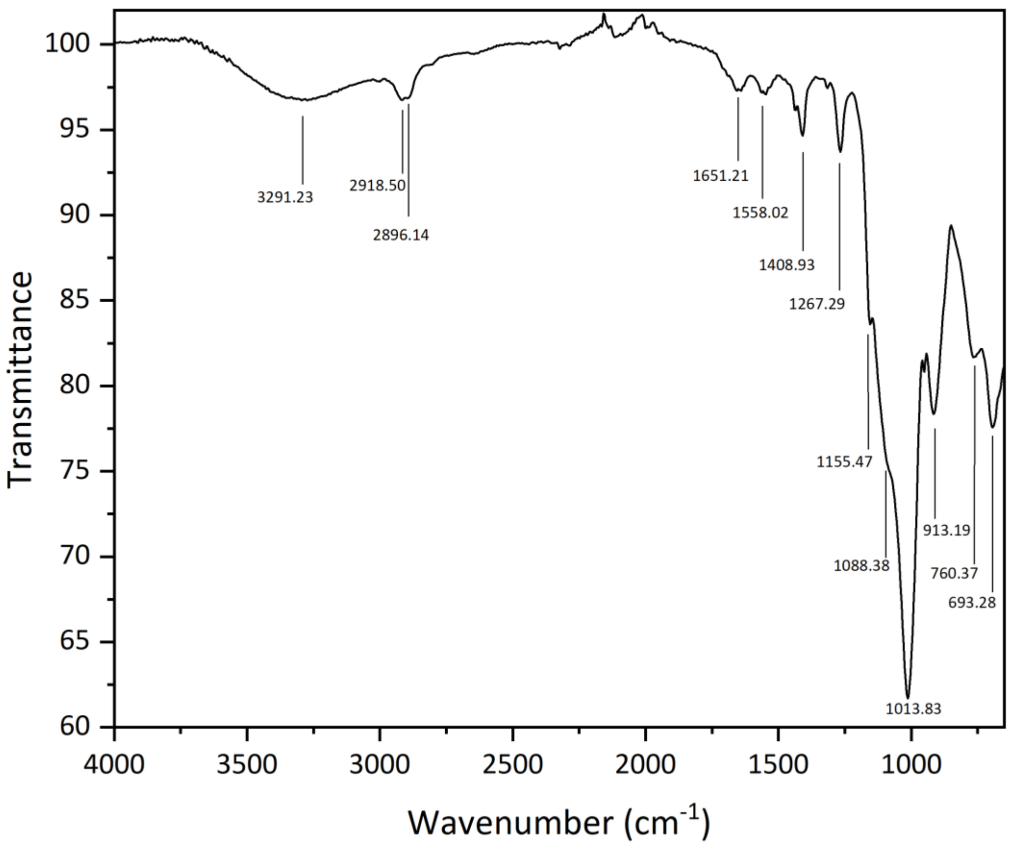

Supplementary Figure S8 IR-spectrum of AVPI@PMO (**SS36**) were measured within the wavenumber range of 4000 to 650 cm^{-1}.

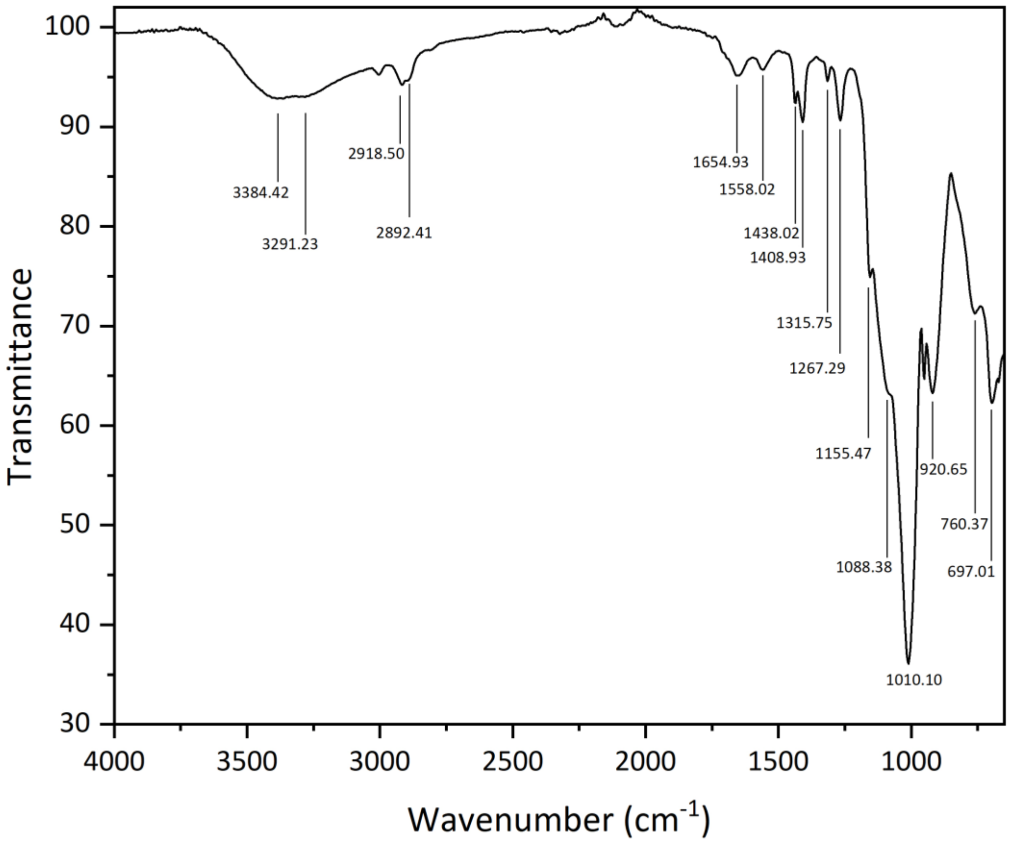

Supplementary Figure S9 Calibration curve of Phenylalanine (λ_{ex} 240nm, λ_{em} 282nm)

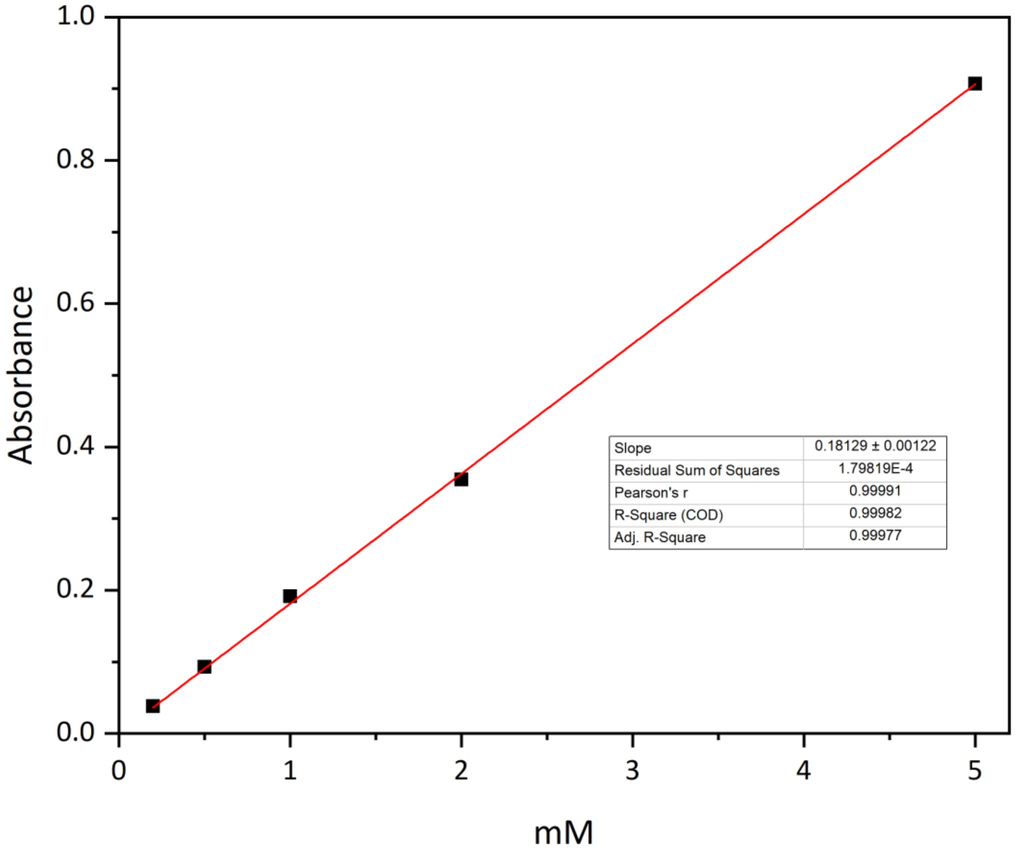

Supplementary Figure S10 Calibration curve of Fmoc-Alanine (λ_{ex} 397nm, λ_{em} 280nm)

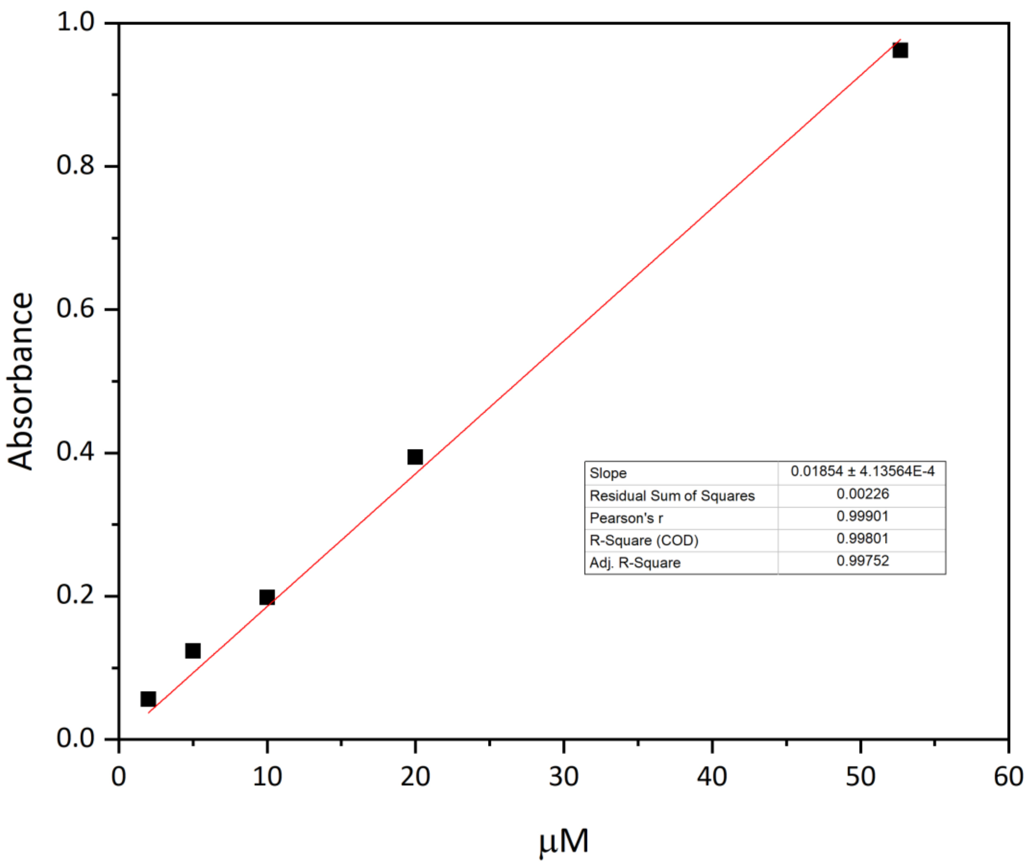

Supplement: Supplementary file 1 [file DataSheet1.pdf]
